# Polymorphisms in *ERAP1* and *ERAP2* Genes Are Associated With Tuberculosis in the Han Chinese

**DOI:** 10.3389/fgene.2020.566190

**Published:** 2020-11-05

**Authors:** Shuqiong Zhang, Shuyuan Liu, Nannan Liu, Chuanyin Li, Hui Wang, Lei Shi, Xinwen Zhang, Ling Bao, Yufeng Yao, Li Shi

**Affiliations:** ^1^Institute of Medical Biology, Chinese Academy of Medical Sciences & Peking Union Medical College, Kunming, China; ^2^Yunnan Key Laboratory of Vaccine Research and Development on Severe Infectious Diseases, Kunming, China; ^3^The Third People’s Hospital of Kunming, Kunming, China

**Keywords:** tuberculosis, *ERAP1* and *ERAP2*, SNPs, association, Han population

## Abstract

Single nucleotide polymorphisms (SNPs) in the endoplasmic reticulum aminopeptidase (*ERAP1* and *ERAP2*) genes are associated with the pathogenesis of bacterial and viral infections. To search for the variations in the *ERAP1* and *ERAP2* genes associated with tuberculosis (TB), 449 TB cases and 435 healthy individuals of the Han population in the Yunnan province of China were included in the present study. Eleven SNPs of *ERAP*s were genotyped using the SNaPshot SNP assay. Allelic, genotypic, and haplotypic association analyses were performed between the TB and control groups. Furthermore, stratification analyses among pulmonary TB (PTB), extrapulmonary TB (EPTB), and healthy controls; and initial treatment TB (ITTB), retreatment TB (RTB), and healthy controls were also performed. The TT genotype of rs26618 in *ERAP1* exhibited a protective factor for TB, compared with the role of the CC/CT genotype (*P* = 0.003; OR = 1.490, 95% CI: 1.140–1.940). In *ERAP2*, the frequency of the G allele of rs2549782 was higher in the case group than in the control group (0.491 vs. 0.417, *P* = 0.002, OR = 1.350, 95% CI: 1.118–1.631), and the TT genotype exhibited a protective factor for TB, compared with the role of the GG/GT genotype (*P* = 0.001; OR = 1.650, 95% CI: 1.230–2.220). The frequency of the C allele of rs1056893 was higher in the case group than in the control group (0.468 vs. 0.394, *P* = 0.002, OR = 1.350, 95% CI: 1.118–1.631), and the genotype exhibited a difference in the log-additive model (*P* = 0.002; OR = 1.350, 95% CI: 1.120–1.630). The frequencies of the haplotype rs27037-rs27044-s30187-rs26618-rs26653-rs3734016-GCCCGC in *ERAP1* (0.290 vs. 0.240, *P-adj* = 0.028, OR = 1.320, 95% CI: 1.063–1.638) and the haplotypes rs2549782-rs2248374-rs2287988-rs1056893-GTAGC in *ERAP2* (0.446 vs. 0.348, *P-adj* = 4.80E-05, OR = 1.510, 95% CI: 1.246–1.829) was higher in the TB groups, while the frequencies of the haplotypes rs2549782-rs2248374-rs2287988-rs1056893-TAGAT (0.478 vs. 0.539, *P-adj* = 0.020, OR = 0.782, 95% CI: 0.649–0.943) were lower in the TB groups. The allelic and genotypic associations were also investigated in the subsequent stratification between the PTB, EPTB and control groups as well as between the ITTB, RTB, and control groups. In conclusion, variations in *ERAP1* and *ERAP2* genes were identified to be associated with TB in the Han Chinese population.

## Highlights

- The variations of rs26618 in *ERAP1*, rs2549782, and rs1056893 in ERAP2 were associated with TB risk in the Han population.
- Four SNPs in *ERAP2*, rs2549782, rs2248374, rs2287988, and rs1056893 were associated with PTB, EPTB, or RTB in the Han population.

## Introduction

Tuberculosis (TB) remains a serious public health problem, being one of the top 10 causes of mortality worldwide as well as the leading cause of mortality from a single infectious agent. In 2018, about 10 million people developed TB, with the disease claiming approximately 1.9 million lives, while India, China, and six other counties accounted for two thirds of the global TB cases (WHO, 2019). TB is caused due to infection by *Mycobacterium tuberculosis* (*M. tb*), which mostly affects the lungs and some extrapulmonary sites and is referred to as pulmonary TB (PTB) and extrapulmonary TB (EPTB), respectively (WHO, 2019). With a high level of exposure, about 80–90% of the population is expected to be infected by *M. tb*, with 5% of the infected individuals developing clinical TB within 2 years of infection, and the remainder developing a latent infection (Abel et al., 2014). Only a small fraction (approximately 5–10%) of the individuals with a latent infection will develop a clinical case of TB throughout their lifetime (Abel et al., 2014; Pai et al., 2016; Dallmann-Sauer et al., 2018).

The mechanism of TB pathogenesis is not clear; however, several studies have suggested that genetic changes, especially in the immune-related genes, may be associated with the development of TB (Fol et al., 2015; van Tong et al., 2017). Immediately after an *M. tb* infection, the host innate immune system initiates the first line of defense. Several genes are involved in this first stage, and polymorphisms in the genes have been reported to be associated with TB, including genes for the Toll-like receptors, the mannose-binding lectin, CD14, surfactant proteins, the dendritic cell-specific ICAM-3-grabbing non-integrin, and the mannose receptor (Fol et al., 2015; van Tong et al., 2017). Subsequently, the adaptive immune response is activated. The genes involved in antigen presentation, induction and function of cytokines/chemokines, and associated receptors have been suggested to play important roles in controlling an *M. tb* infection (Fol et al., 2015; Mao et al., 2015). In recent genome-wide association studies (GWAS) of TB risk in the Han Chinese population, new risk loci in *ASAP1*, *ESRRB*, and *TGM6* genes have been identified (Chen et al., 2016a; Zheng et al., 2018).

During the process of antigen presentation by the major histocompatibility complex (MHC) class I, the endoplasmic reticulum aminopeptidases (ERAP) trim peptides to the optimal size for MHC-I binding, to ensure the correct assembly of the peptide-loading complex (PLC), which plays an important role in inducing the correct immune response against pathogens (Chang et al., 2005; Chen et al., 2016b). ERAPs, including ERAP1 and ERAP2, were initially identified as homologs of the human placental leucine aminopeptidase or the insulin-regulated aminopeptidase, and they belong to the oxytocinase subfamily of the M1 zinc metallopeptidase family (Serwold et al., 2002). The human *ERAP* genes are located on chromosome 5q15 in opposite orientation; *ERAP1* is 47,379 bp in length and comprises 20 exons, and *ERAP2* gene is 41,438 bp in length and comprises 19 exons (Hattori et al., 2001; Hattori and Tsujimoto, 2013). As the human leukocyte antigen (HLA) class I gene, *ERAP* also presents diverse polymorphisms, which have been shown to affect several infectious disease processes. For example, the rs2549782 of the *ERAP2* gene is associated with the human immunodeficiency virus (HIV), and the TT genotype is over-represented in the HIV-1-exposed seronegative group (Cagliani et al., 2010). The rs2549782 was identified as a risk factor in a Spanish population exposed to HIV-1 infection by intravenous drug administration (Biasin et al., 2013). In our previous study, we found that the A allele of rs2248374 (located in the splice/intron region), compared with the G allele, increased the risk for developing a hepatitis C virus (HCV) chronic infection (Liu et al., 2017).

Some studies have shown that HLA alleles (*HLA-A^∗^02:01, HLA*-*A^∗^11:01*, and *HLA-B^∗^57*) increase the susceptibility or provide resistance to TB; however, the influence of ERAP alleles on TB has not been examined (Geluk et al., 2000; Liu et al., 2016; Petros et al., 2017). In the present study, we genotyped single nucleotide polymorphisms in the *ERAP1* and *ERAP2* genes in TB patients to determine if the *ERAP* gene variants are associated with *M. tb* infection and TB progression.

## Materials and Methods

### Subjects

The present study included a total of 449 cases from the Third Hospital of Kunming admitted between 2018 and 2019 with latent TB and 435 cases of unrelated healthy Chinese Han individuals as controls from the Yunnan province (southwest China). The cases were divided into pulmonary TB (PTB) and extrapulmonary TB (EPTB) groups, with EPTB defined as TB affecting the extrapulmonary sites, such as the lymph nodes, abdomen, urinary tract, skin, joints, bones, and meninges, exclusively or in combination with PTB. Additionally, the patients with TB were stratified into initial treated TB (ITTB) and re-treated TB (RTB) according to their treatment history. The ITTB were defined as patients who never received anti-TB drug therapy, or those who underwent anti-TB drug therapy but did not complete the treatment, or those who received unstandardized treatment for no more than 1 month, while the RTB were defined as patients who underwent unstandardized treatment within 1 month of diagnosis, or those who failed to respond to initial treatment and reported recurrent TB (WS 196-2017) (China National Health and Family Planning Commission of the People’s Republic of China, 2018a). All experimental protocols used in this study were approved by the Institutional Review Board of the Third Hospital of Kunming. All procedures were in accordance with the guidelines and principles of the Declaration of Helsinki and its later amendments or comparable ethical standards (General Assembly of the World Medical Association, 2014). All participants provided written informed consent.

Diagnoses of TB were based on the standard case definitions for TB reported by the World Health Organization (WHO) and the Diagnosis for Pulmonary Tuberculosis (WS 288-2017) (China National Health and Family Planning Commission of the People’s Republic of China, 2018b) and the Classification of Tuberculosis (WS 196-2017) (China National Health and Family Planning Commission of the People’s Republic of China, 2018a) from the Health Industry Standard of the People’s Republic of China. The diagnostic criteria were as follows: (1) *Mycobacterium tuberculosis* infection confirmed by bacteriological assessment such as the tuberculin skin test, an interferon-γ release assay, and a culture-positive sputum smear; and (2) clinical symptoms and chest X-ray consistent with TB. Patients with immunodeficiency, autoimmune diseases, or other acute or chronic infections were excluded from this study. All controls had a negative TB disease history and had no acute or chronic pulmonary disease, bacterial or viral infection, or other immune-mediated disorders.

### SNP Selection and Genotyping

Six SNPs (rs27037, rs27044, rs30187, rs26618, rs26653, and rs3734016) located in *ERAP1* and five SNPs (rs2549782, rs2548538, rs2248374, rs2287988, and rs1056893) located in *ERAP2* were selected. All the SNPs exhibited variant polymorphisms with minor allele frequencies within the range of 16.0 to 49.5%.

Approximately 2–5 mL of the peripheral blood was drawn from each participant, and genomic DNA was extracted from the peripheral lymphocytes using the QIAamp Blood Mini Kit (Qiagen, Hilden, Germany). Genotyping of the total 11 SNPs was performed using the SNaPshot SNP assay (Thermo Fisher Scientific, Waltham, MA, United States) according to a previous study (Li et al., 2020). Briefly, multiplex polymerase chain reaction (PCR) of the SNPs was performed followed by a single-nucleotide primer extension assay test. The PCR application primers and the extension primers are shown in Supplementary Tables 1, 2. The 11 SNPs were divided into three groups and detected by capillary electrophoresis (Supplementary Figures 1–3), followed by analysis using GeneMapper^TM^ 4.0 software (Applied Biosystems, Foster City, CA, United States). For quality control, 5% of the samples from the case and control groups were genotyped twice and denoted with unique serial numbers of analysis, and the reproducibility was found to be 100%. Furthermore, we sequenced each SNP in the pre-experiment, and selected all the ambiguous samples for sequencing to confirm the result (Supplementary Figure 4).

### Statistical Analysis

The Hardy–Weinberg equilibrium (HWE) was evaluated to determine the representativeness of the study population. Age and sex distribution comparisons between the case and control groups were determined by the *t*-test and the Chi-squared (χ^2^) test using SPSS (version 19.0; SPSS Inc., Chicago, IL, United States). Allelic and genotypic frequencies of these SNPs were compared between different groups using the Chi-squared test, and odds ratios (ORs) and associated 95% confidence intervals (CIs) were calculated using the SHEsis software (Shi and He, 2005; Li et al., 2009). Five inheritance models (codominant, dominant, recessive, overdominant, and log-additive) were analyzed. Simultaneously, the Akaike information criterion (AIC) and Bayesian information criterion (BIC) values were calculated to determine the inheritance model with the best fit, i.e., the model with the smallest AIC and BIC values (Sole et al., 2006). Additionally, linkage disequilibrium (LD) was calculated and a D’ value greater than 0.80 was considered to indicate LD. The haplotypes among these SNPs were analyzed using SHEsis software (Shi and He, 2005; Li et al., 2009). *P* < 0.05 was considered statistically significant, with Bonferroni’s corrections for multiple testing calculated as 0.05/n.

## Results

### Characteristics of the Subjects

Table 1 shows the clinical data of the subjects in the present study. There was no significant difference in age and sex between the TB and control groups (*P* > 0.05). The TB group was further divided into the PTB and EPTB groups, and the average age of the EPTB group was higher than that of the control group (39.75 ± 15.86 vs. 45.32 ± 8.99, *P* = 0.002). In the TB group, 278 and 171 cases belonged to the ITTB and RTB groups, respectively. The average age of the ITTB group was lower than that of the control group (43.12 ± 16.31 vs. 45.32 ± 8.99, *P* = 0.039), and the male to female ratio in the RTB group was 1.59:1, while that in the ITTB group was 1.07:1 (*P* = 0.047).

**TABLE 1:** Demographics and clinical data for tuberculosis patients and controls.

**Study groups**	**Num. of cases**	**Sex, M:F**	**Mean age**
TB	449	249: 200	43.71 ± 15.99
PTB	324	190: 134	45.29 ± 15.84
Infiltration	145	80: 65	45.82 ± 16.89
Secondary	161	106: 55	44.50 ± 14.80
Cavity	18	4: 14	47.17 ± 15.99
EPTB	125	59: 66	39.75 ± 15.86
Lymph nodes	13	7: 6	36.31 ± 13.68
Genitourinary tract	31	7: 24	40.48 ± 14.66
Bone and joint	24	13: 11	41.58 ± 15.20
Cutaneous	5	1: 4	42.20 ± 21.811
Celiac	14	8: 6	41.00 ± 23.29
Meningitis	8	8: 0	34.13 ± 10.87
Peritoneal	2	1: 1	20.50 ± 6.36
Pleuritis	1	0: 1	47.00
Multi-site concurrency	17	8: 9	39.53 ± 13.01
Others	10	6: 4	42.60 ± 18.91
ITTB	278	144: 134	43.12 ± 16.31
RTB	171	105: 66	44.68 ± 15.46
Health control	435	238: 197	45.32 ± 8.99

*M, male; F, female; TB, tuberculosis; PTB, pulmonary TB; EPTB, extrapulmonary TB; ITTB, initial treatment TB; RTB, retreatment TB.*

### Comparison of Allelic and Genotypic Frequencies of *ERAP1* and *ERAP2* Between TB Patients and Controls

All 11 SNPs were in HWE in both the control and TB case groups (*P* > 0.05) (Supplementary Table 3). The allelic and genotypic frequencies of these SNPs are presented in Table 2. The rs2549782 polymorphism in *ERAP2* differed between the TB and healthy control groups at both the allelic and genotypic levels. The frequency of the G allele was higher in the case group than in the control group (0.491 vs. 0.417, *P* = 0.002, OR = 1.348, 95% CI: 1.117–1.626). The rs1056893 polymorphism in *ERAP2* also differed between the TB and healthy control groups at the allelic level, but showed a trend difference only at the genotypic level. The frequency of the C allele was higher in the case group than in the control group (0.468 vs. 0.394, *P* = 0.002, OR = 1.350, 95% CI: 1.118–1.631). The other three SNPs in *EARP2* including rs2548538, rs2248374, and rs2287988, showed a trend of having different allelic frequencies, but they were not significantly different after the Bonferroni correction. Similarly, the rs26618 and rs3734016 SNPs in *ERAP1* showed different allelic and genotypic frequencies, but they were not significantly different after the Bonferroni correction.

**TABLE 2:** The allelic and genotypic distribution of ERAP1 and ERAP2 genes in the TB and the control groups.

**Gene**	**SNP**	**Genotype**	**Control**	**Case**	***P*-value**	**OR (95% CI)**
			**n (freq.)**	**n (freq.)**		
ERAP1	rs27037	G	508 (0.584)	517 (0.576)	0.727	1.034 (0.856–1.249)
		T	362 (0.416)	381 (0.424)		
		G/G	149 (0.343)	155 (0.345)	0.719	
		G/T	210 (0.483)	207 (0.461)		
		T/T	76 (0.175)	87 (0.194)		
	rs27044	C	457 (0.525)	496 (0.552)	0.254	0.897 (0.744–1.081)
		G	413 (0.475)	402 (0.448)		
		C/C	116 (0.267)	131 (0.292)	0.490	
		C/G	225 (0.517)	234 (0.521)		
		G/G	94 (0.216)	84 (0.187)		
	rs30187	C	431 (0.495)	473 (0.527)	0.188	0.882 (0.732–1.063)
		T	439 (0.505)	425 (0.473)		
		C/C	108 (0.248)	127 (0.283)	0.421	
		C/T	215 (0.494)	219 (0.488)		
		T/T	112 (0.257)	103 (0.229)		
	rs26618	T	641 (0.737)	614 (0.684)	0.014	1.295 (1.053–1.591)
		C	229 (0.263)	284 (0.316)		
		T/T	238 (0.547)	201 (0.448)	0.011	
		C/T	165 (0.379)	212 (0.472)		
		C/C	32 (0.074)	36 (0.080)		
	rs26653	C	464 (0.533)	464 (0.517)	0.484	1.069 (0.887–1.288)
		G	406 (0.467)	434 (0.483)		
		C/C	127 (0.292)	111 (0.247)	0.208	
		G/C	210 (0.483)	242 (0.539)		
		G/G	98 (0.225)	96 (0.214)		
	rs3734016	C	731 (0.840)	785 (0.874)	0.041	0.757 (0.579–0.990)
		T	139 (0.160)	113 (0.126)		
		C/C	306 (0.703)	347 (0.773)	0.047	
		C/T	119 (0.274)	91 (0.203)		
		T/T	10 (0.023)	11 (0.024)		
ERAP2	rs2549782	T	507 (0.583)	457 (0.509)	**0.002**	**1.348 (1.117–1.626)**
		G	363 (0.417)	441 (0.491)		
		T/T	147 (0.338)	106 (0.236)	0.003	
		T/G	213 (0.490)	245 (0.546)		
		G/G	75 (0.172)	98 (0.218)		
	rs2548538	A	502 (0.577)	468 (0.521)	0.018	1.253 (1.039–1.512)
		T	368 (0.423)	430 (0.479)		
		A/A	142 (0.326)	120 (0.267)	0.058	
		A/T	218 (0.501)	228 (0.508)		
		T/T	75 (0.172)	101 (0.225)		
	rs2248374	G	507 (0.583)	476 (0.530)	0.026	1.238 (1.026–1.494)
		A	363 (0.417)	422 (0.470)		
		G/G	149 (0.343)	125 (0.278)	0.080	
		G/A	209 (0.480)	226 (0.503)		
		A/A	77 (0.177)	98 (0.218)		
	rs2287988	A	515 (0.592)	473 (0.527)	0.006	1.303 (1.080–1.574)
		G	355 (0.408)	425 (0.473)		
		A/A	150 (0.345)	119 (0.265)	0.018	
		A/G	215 (0.494)	235 (0.523)		
		G/G	70 (0.161)	95 (0.212)		
	rs1056893	T	527 (0.606)	478 (0.532)	**0.002**	**1.350 (1.118–1.631)**
		C	343 (0.394)	420 (0.468)		
		T/T	161 (0.370)	127 (0.283)	0.008	
		C/T	205 (0.471)	224 (0.499)		
		C/C	69 (0.159)	98 (0.218)		

*The statistical significant threshold was set at *P* < 0.0045 after Bonferroni correction. The significant association after Bonerroni correction were indicated in bold.*

### Inheritance Model Analysis

To evaluate the genotypic association of the 11 SNPs with TB, an inheritance analysis was performed (Table 3). The comparison between the TB and control groups indicated that rs26618 and rs2549782 exhibited different frequencies in the dominant model, the best-fit inheritance model. The TT genotype of rs26618 was a protective factor for TB, compared with the role of the CC/CT genotype (*P* = 0.003; OR = 1.490, 95% CI: 1.140–1.940). The TT genotype of rs2549782 was also a protective factor for TB, compared with the role of the GG/GT genotype (*P* = 0.001; OR = 1.650, 95% CI: 1.230–2.220). Four SNPs rs1056893, rs2548538, rs2248374, and rs2287988, exhibited different trends in the log-additive model, the best-fit inheritance model. However, the frequency difference was significant only in rs1056893 after the Bonferroni correction (*P* = 0.002; OR = 1.350, 95% CI: 1.120–1.630).

**TABLE 3:** The best-fit inheritance models analysis for each SNPs in ERAP1 and ERAP2 gene between the TB and the control groups.

**SNP**	**Model**	**Genotype**	**Control**	**Case**	***P*-value**	**OR (95% CI)**	**AIC**	**BIC**
			**n (freq.)**	**n (freq.)**				
rs27037	Recessive	G/G-G/T	359 (0.825)	362 (0.806)		Ref	1228.7	1238.3
		T/T	76 (0.175)	87 (0.194)	0.470	1.140 (0.810–1.600)		
rs27044	Log-additive	–	–	–	0.240	0.890 (0.740–1.080)	1227.9	1237.5
rs30187	Log-additive	–	–	–	0.190	0.880 (0.730–1.060)	1227.6	1237.1
rs26618	Dominant	T/T	238 (0.547)	201 (0.448)		Ref	1220.5	1230.1
		C/T-C/C	197 (0.453)	248 (0.552)	0.003	1.490 (1.140–1.940)		
rs26653	Overdominant	C/C-G/G	225 (0.517)	207 (0.461)		Ref	1226.5	1236
		G/C	210 (0.483)	242 (0.539)	0.094	1.250 (0.960–1.630)		
rs3734016	Overdominant	C/C-T/T	316 (0.726)	358 (0.797)		Ref	1223.1	1232.7
		C/T	119 (0.274)	91 (0.203)	0.013	0.670 (0.490–0.920)		
rs2549782	Log-additive	–	–	–	0.001	1.370 (1.130–1.660)	1219	1228.6
rs2548538	Recessive	A/A-A/T	360 (0.828)	348 (0.775)		Ref	1225.4	1235
		T/T	75 (0.172)	101 (0.225)	0.050	1.390 (1.000–1.940)		
rs2248374	Log-additive	–	–	–	0.026	1.240 (1.030–1.490)	1224.3	1233.9
rs2287988	Log-additive	–	–	–	0.005	1.320 (1.090–1.600)	1221.4	1230.9
rs1056893	Log-additive	–	–	–	0.002	1.350 (1.120–1.630)	1219.6	1229.2

*The statistical significant threshold was set at *P* < 0.0045 after Bonferroni correction.AIC, Akaike information criterion; BIC, Bayesian information criterion.*

### LD and Haplotype Analysis of SNPs in *ERAP1* and *ERAP2*

**FIGURE 1:**
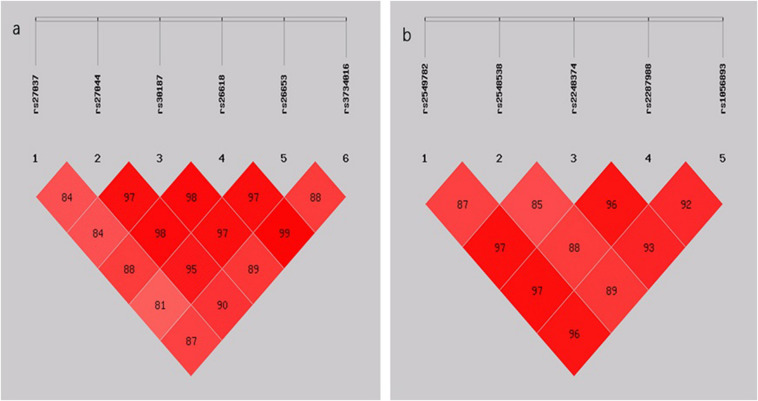
**(A)** LD pattern of the five SNPs genotyped in the *ERAP1* gene; **(B)** LD pattern of the five SNPs genotyped in the *ERAP2* gene. The plots show D’ × 100 values as a pair of LD.

The LD analysis showed that all six SNPs in *ERAP1* and five SNPs in *ERAP2* were in LD (*D’* > 0.80) (Figure 1). Subsequently, the haplotypes of rs27037-rs27044-s30187-rs26618-rs26653-rs3734016 and rs2549782-rs2248374-rs2287988-rs1056893 were constructed. The distribution of these haplotypes (with a frequency of more than 3%) was compared in a pairwise manner between the TB and control groups (Table 4). The frequencies of the haplotype rs27037-rs27044-s30187-rs26618-rs26653-rs3734016-GCCCGC in *ERAP1* was higher in the TB group (0.290 vs. 0.240, *P-adj* = 0.028, OR = 1.320, 95% CI: 1.063–1.638). The frequencies of the haplotype rs2549782-rs2248374-rs2287988-rs1056893-GTAGC in *ERAP2* (0.446 vs. 0.348, *P-adj* = 4.80E-045, OR = 1.510, 95% CI: 1.246–1.829) was higher in the TB group, while the frequencies of the haplotype rs2549782-rs2248374-rs2287988-rs1056893-TAGAT (0.478 vs. 0.539, *P-adj* = 0.020, OR = 0.782, 95% CI: 0.649–0.943) was lower in the TB group.

**TABLE 4:** The major ERAP1 and ERAP2 haplotypes in the TB and the case groups.

**Haplotypes**	**Control n (freq)**	**Case n (freq)**	**x2**	***P*-value**	***P*_adj.**	**OR (95% CI)**
rs27037-rs27044-s30187-rs26618-rs26653-rs3734016						
G C C C G C	209 (0.240)	261 (0.290)	6.359	0.012	0.028	1.320 (1.063–1.638)
G C C T G C	41 (0.048)	37 (0.041)	0.362	0.548	>0.05	0.870 (0.553–1.370)
G C C T G T	120 (0.138)	103 (0.115)	2.151	0.142	>0.05	0.809 (0.610–1.074)
G C T T C C	23 (0.027)	37 (0.041)	2.806	0.094	>0.05	1.563 (0.923–2.647)
G G T T C C	74 (0.085)	50 (0.055)	5.762	0.016	>0.05	0.634 (0.436–0.922)
T G T T C C	327 (0.376)	328 (0.366)	0.149	0.699	>0.05	0.962 (0.788–1.173)
rs2549782-rs2248374-rs2287988-rs1056893						
G T A G C	303 (0.348)	401 (0.446)	17.81	2.40E-05	4.80E-05	1.510 (1.246–1.829)
T A G A T	469 (0.539)	429 (0.478)	6.655	0.010	0.020	0.782 (0.649–0.943)

### Stratification Analysis of the Association Between TB and ERAP Polymorphisms

The stratification analysis between the PTB, EPTB, and control groups was performed at first, and the *P*-value, OR, and 95% CI of the pair-wise comparison between the EPTB and the control group were calculated on the basis of the logistic regression model adjusted by age (Table 5 and Supplementary Table 4). The rs2549782 polymorphism *in ERAP2* differed between the PTB and control groups at both the allelic and genotypic levels; however, it did not differ between the EPTB and control groups. The frequency of the G allele was higher in the PTB than in the control group (0.494 vs. 0.417, *P* = 0.003, OR = 1.363, 95% CI: 1.110–1.672), and the GT/GG genotype was a resistant factor for PTB, compared with the role of the TT genotype (*P* = 4.00E-04; OR = 1.790, 95% CI: 1.290–2.480) in the dominant model. Genotypic differences were observed in the log-additive model of rs2548538 between the EPTB and control groups (*P* = 0.001, OR = 1.650, 95% CI: 1.220–2.220), but not between the PTB and control groups.

**TABLE 5:** The allelic and genotype distribution of ERAP1 and ERAP2 genes among the control, PTB and EPTB groups.

**Gene**	**SNP**	**Genotype**	**Control**	**PTB**	**EPTB**	**PTB vs. Control**		EPTB vs. Control*	
			**n (freq.)**	**n (freq.)**	**n (freq.)**	***P*-value**	**OR (95% CI)**	***P*-value**	**OR (95% CI)**
ERAP1	rs27037	G	508 (0.584)	380 (0.586)	138 (0.552)	0.922	0.990 (0.805–1.217)	0.530	1.098 (0.820–1.470)
		T	362 (0.416)	268 (0.414)	112 (0.448)				
		G/G	149 (0.343)	113 (0.349)	43 (0.344)	0.978		0.340	
		G/T	210 (0.483)	154 (0.475)	52 (0.416)				
		T/T	76 (0.175)	57 (0.176)	30 (0.240)				
	rs27044	C	457 (0.525)	354 (0.546)	143 (0.572)	0.417	0.919 (0.749–1.127)	0.101	0.783 (0.585–1.049)
		G	413 (0.475)	294 (0.454)	107 (0.428)				
		C/C	116 (0.267)	92 (0.284)	40 (0.320)	0.679		0.240	
		C/G	225 (0.517)	170 (0.525)	63 (0.504)				
		G/G	94 (0.216)	62 (0.191)	22 (0.176)				
	rs30187	C	431 (0.495)	340 (0.525)	134 (0.536)	0.259	0.889 (0.726–1.090)	0.156	0.811 (0.606–1.084)
		T	439 (0.505)	308 (0.475)	116 (0.464)				
		C/C	108 (0.248)	91 (0.281)	36 (0.288)	0.528		0.370	
		C/T	215 (0.494)	158 (0.488)	62 (0.496)				
		T/T	112 (0.257)	75 (0.231)	27 (0.216)				
	rs26618	T	641 (0.737)	442 (0.682)	171 (0.684)	0.02	1.305 (1.043–1.632)	0.084	1.320 (0.963–1.809)
		C	229 (0.263)	206 (0.318)	79 (0.316)				
		T/T	238 (0.547)	145 (0.448)	55 (0.440)	0.024		0.063	
		C/T	165 (0.379)	152 (0.469)	61 (0.488)				
		C/C	32 (0.074)	27 (0.083)	9 (0.072)				
	rs26653	C	464 (0.533)	341 (0.526)	122 (0.488)	0.784	1.029 (0.839–1.261)	0.143	1.242 (0.930–1.659)
		G	406 (0.467)	307 (0.474)	128 (0.512)				
		C/C	127 (0.292)	82 (0.253)	28 (0.224)	0.221		0.210	
		G/C	210 (0.483)	177 (0.546)	66 (0.528)				
		G/G	98 (0.225)	65 (0.201)	31 (0.248)				
	rs3734016	C	731 (0.840)	566 (0.873)	219 (0.876)	0.069	0.762 (0.568–1.022)	0.255	0.780 (0.509–1.196)
		T	139 (0.160)	82 (0.127)	31 (0.124)				
		C/C	306 (0.703)	251 (0.775)	96 (0.768)	0.052		0.350	
		C/T	119 (0.274)	64 (0.198)	27 (0.216)				
		T/T	10 (0.023)	9 (0.028)	2 (0.016)				
ERAP2	rs2549782	T	507 (0.583)	328 (0.506)	129 (0.516)	0.003	1.363 (1.110–1.672)	0.066	1.314 (0.983–1.756)
		G	363 (0.417)	320 (0.494)	121 (0.484)				
		T/T	147 (0.338)	72 (0.222)	34 (0.272)	0.002		0.180	
		T/G	213 (0.490)	184 (0.568)	61 (0.488)				
		G/G	75 (0.172)	68 (0.210)	30 (0.240)				
	rs2548538	A	502 (0.577)	352 (0.543)	116 (0.464)	0.189	1.147 (0.935–1.408)	0.001	1.617 (1.208–2.163)
		T	368 (0.423)	296 (0.457)	134 (0.536)				
		A/A	142 (0.326)	95 (0.293)	25 (0.200)	0.401		0.004	
		A/T	218 (0.501)	162 (0.500)	66 (0.528)				
		T/T	75 (0.172)	67 (0.207)	34 (0.272)				
	rs2248374	G	507 (0.583)	348 (0.537)	128 (0.512)	0.076	1.204 (0.981–1.478)	0.047	1.342 (1.004–1.795)
		A	363 (0.417)	300 (0.463)	122 (0.488)				
		G/G	149 (0.343)	92 (0.284)	33 (0.264)	0.192		0.140	
		G/A	209 (0.480)	164 (0.506)	62 (0.496)				
		A/A	77 (0.177)	68 (0.210)	30 (0.240)				
	rs2287988	A	515 (0.592)	345 (0.532)	128 (0.512)	0.021	1.274 (1.038–1.564)	0.026	1.392 (1.041–1.861)
		G	355 (0.408)	303 (0.468)	122 (0.488)				
		A/A	150 (0.345)	86 (0.265)	33 (0.264)	0.050		0.079	
		A/G	215 (0.494)	173 (0.534)	62 (0.496)				
		G/G	70 (0.161)	65 (0.201)	30 (0.240)				
	rs1056893	T	527 (0.606)	352 (0.543)	126 (0.504)	0.015	1.292 (1.052–1.587)	0.004	1.531 (1.144–2.048)
		C	343 (0.394)	296 (0.457)	124 (0.496)				
		T/T	161 (0.370)	95 (0.293)	32 (0.256)	0.050		0.018	
		C/T	205 (0.471)	162 (0.500)	62 (0.496)				
		C/C	69 (0.159)	67 (0.207)	31 (0.248)				

The statistical significant threshold was set at *P* < 0.0045 after Bonferroni correction.*The *P*-value, OR and 95% CIs of pairs comparison between PTB and control and EPTB and control were calculated on the basis of the logistic regression model adjusted by age.

Next, stratification analysis between the ITTB and RTB groups was performed (Table 6 and Supplementary Table 5). The *P*-values, ORs, and 95% CIs of the pair-wise comparisons between ITTB and control, and between RTB and control were calculated on the basis of the logistic regression model adjusted by age and sex. Notably, all five SNPs in *ERAP2* exhibited differences both at allelic and genotypic levels between the RTB and control groups; however, only the CT genotype of rs26618 in *ERAP1* was a protective factor compared with the role of the CC/TT genotype in the Overdominant Model between the ITTB and control groups (0.489 vs. 0.379, *P* = 0.004, OR = 1.570, 95% CI: 1.160–2.130). The minor alleles of all five SNPs were found to be the risk factors in the RTB group compared with those in the control group, and the genotypic differences in the log-additive model of all five SNPs were significant. In addition, the frequencies of the haplotype rs2549782-rs2248374-rs2287988-rs1056893-GTAGC were higher in the RTB group than in the control group (0.500 vs. 0.348, *P* = 2.00E-06, OR = 1.871, 95% CI: 1.452–2.412), while the frequencies of the haplotype rs2549782-rs2248374-rs2287988-rs1056893-TAGAT were lower in the RTB group than in the control group (0.418 vs. 0.539, *P* = 3.00E-04, OR = 0.614, 95% CI: 0.477–0.791).

**TABLE 6:** The allelic and genotype distribution of ERAP1 and ERAP2 genes among the control, PTB and EPTB groups.

**Gene**	**SNP**	**Genotype**	**Control**	**ITTB**	**RTB**	ITTB vs. Control*		**RTB vs. Control**		RTB vs. ITTB**	
			**n (freq.)**	**n (freq.)**	**n (freq.)**	***P*-value**	**OR (95% CI)**	***P*-value**	**OR (95% CI)**	***P*-value**	**OR (95% CI)**
ERAP1	rs27037	G	508 (0.584)	314 (0.565)	203 (0.594)	0.464	1.084 (0.873–1.346)	0.759	0.961 (0.745–1.239)	0.341	0.875 (0.665–1.152)
		T	362 (0.416)	242 (0.435)	139 (0.406)						
		G/G	149 (0.343)	89 (0.320)	66 (0.386)	0.740		0.324		0.230	
		G/T	210 (0.483)	136 (0.489)	71 (0.415)						
		T/T	76 (0.175)	53 (0.191)	34 (0.199)						
	rs27044	C	457 (0.525)	308 (0.554)	188 (0.550)	0.279	0.888 (0.716–1.101)	0.443	0.906 (0.705–1.165)	0.954	1.008 (0.768–1.324)
		G	413 (0.475)	248 (0.446)	154 (0.450)						
		C/C	116 (0.267)	75 (0.270)	56 (0.327)	0.140		0.224		0.031	
		C/G	225 (0.517)	158 (0.568)	76 (0.444)						
		G/G	94 (0.216)	45 (0.162)	39 (0.228)						
	rs30187	C	431 (0.495)	289 (0.520)	184 (0.538)	0.377	0.908 (0.733–1.125)	0.182	0.843 (0.656–1.083)	0.56	0.922 (0.703–1.210)
		T	439 (0.505)	267 (0.480)	158 (0.462)						
		C/C	108 (0.248)	72 (0.259)	55 (0.322)	0.510		0.174		0.140	
		C/T	215 (0.494)	145 (0.522)	74 (0.433)						
		T/T	112 (0.257)	61 (0.219)	42 (0.246)						
	rs26618	T	641 (0.737)	380 (0.683)	234 (0.684)	0.029	1.300 (1.028–1.645)	0.066	1.292 (0.983–1.698)	0.973	0.995 (0.743–1.331)
		C	229 (0.263)	176 (0.317)	108 (0.316)						
		T/T	238 (0.547)	122 (0.439)	79 (0.462)	0.012		0.163		0.560	
		C/T	165 (0.379)	136 (0.489)	76 (0.444)						
		C/C	32 (0.074)	20 (0.072)	16 (0.094)						
	rs26653	C	464 (0.533)	290 (0.522)	174 (0.509)	0.618	1.056 (0.852–1.308)	0.441	1.103 (0.859–1.417)	0.705	1.054 (0.804–1.382)
		G	406 (0.467)	266 (0.478)	168 (0.491)						
		C/C	127 (0.292)	69 (0.248)	42 (0.246)	0.210		0.492		0.820	
		G/C	210 (0.483)	152 (0.547)	90 (0.526)						
		G/G	98 (0.225)	57 (0.205)	39 (0.228)						
	rs3734016	C	731 (0.840)	486 (0.874)	299 (0.874)	0.091	0.765 (0.561–1.044)	0.135	0.756 (0.524–1.092)	0.973	0.993 (0.660–1.494)
		T	139 (0.160)	70 (0.126)	43 (0.126)						
		C/C	306 (0.703)	214 (0.770)	133 (0.778)	0.150		0.116		0.900	
		C/T	119 (0.274)	58 (0.209)	33 (0.193)						
		T/T	10 (0.023)	6 (0.022)	5 (0.029)						
ERAP2	rs2549782	T	507 (0.583)	302 (0.543)	155 (0.453)	0.152	1.171 (0.944–1.452)	4.61E-05	1.685 (1.310–2.168)	0.005	1.472 (1.120–1.933)
		G	363 (0.417)	254 (0.457)	187 (0.547)						
		T/T	147 (0.338)	75 (0.270)	31 (0.181)	0.140		1.69E-04		0.013	
		T/G	213 (0.490)	152 (0.547)	93 (0.544)						
		G/G	75 (0.172)	51 (0.183)	47 (0.275)						
	rs2548538	A	502 (0.577)	313 (0.563)	155 (0.453)	0.62	1.056 (0.851–1.312)	9.99E-05	1.646 (1.279–2.117)	0.001	1.623 (1.234–2.135)
		T	368 (0.423)	243 (0.437)	187 (0.547)						
		A/A	142 (0.326)	87 (0.313)	33 (0.193)	0.880		4.36E-04		0.002	
		A/T	218 (0.501)	139 (0.500)	89 (0.520)						
		T/T	75 (0.172)	52 (0.187)	49 (0.287)						
	rs2248374	G	507 (0.583)	315 (0.567)	161 (0.471)	0.584	1.062 (0.856–1.319)	0.000421	1.570 (1.221–2.019)	0.003	1.520 (1.157–1.997)
		A	363 (0.417)	241 (0.433)	181 (0.529)						
		G/G	149 (0.343)	88 (0.317)	37 (0.216)	0.770		0.002		0.010	
		G/A	209 (0.480)	139 (0.500)	87 (0.509)						
		A/A	77 (0.177)	51 (0.183)	47 (0.275)						
	rs2287988	A	515 (0.592)	312 (0.561)	161 (0.471)	0.286	1.125 (0.906–1.397)	0.000133	1.631 (1.268–2.098)	0.005	1.476 (1.123–1.938)
		G	355 (0.408)	244 (0.439)	181 (0.529)						
		A/A	150 (0.345)	83 (0.299)	36 (0.211)	0.450		6.00E-05		0.015	
		A/G	215 (0.494)	146 (0.525)	89 (0.520)						
		G/G	70 (0.161)	49 (0.176)	46 (0.269)						
	rs1056893	T	527 (0.606)	314 (0.565)	164 (0.480)	0.129	1.183 (0.952–1.469)	6.55E-05	1.668 (1.296–2.145)	0.008	1.448 (1.102–1.901)
		C	343 (0.394)	242 (0.435)	178 (0.520)						
		T/T	161 (0.370)	88 (0.317)	39 (0.228)	0.290		4.01E-04		0.028	
		C/T	205 (0.471)	138 (0.496)	86 (0.503)						
		C/C	69 (0.159)	52 (0.187)	46 (0.269)						

The statistical significant threshold was set at *P* < 0.0045 after Bonferroni correction.*The *P*-value, OR and 95% CIs of pairs comparison between ITTB and control were calculated on the basis of the logistic regression model adjusted by age.**The *P*-value, OR and 95% CIs of pairs comparison between ITTB and RTB were calculated on the basis of the logistic regression model adjusted by gender.

Four SNPs, namely rs2548538, rs2248374, rs2549782, and rs2287988, exhibited differences between the RTB and ITTB groups at either the allelic or genotype level. For rs2548538, the T allele frequencies were higher in the RTB group than in the ITTB group (0.547 vs. 0.473, *P* = 0.001, OR = 1.623, 95% CI: 1.234–2.135), and the genotypic differences were also observed in the log-additive model (*P* = 4.00E-04, OR = 1.650, 95% CI: 1.240–2.190). For rs2248374, the A allele frequencies were higher in the RTB group than in the ITTB group (0.529 vs. 0.433, *P* = 0.003, OR = 1.520, 95% CI: 1.157–1.997), and the genotypic difference was also observed in the log-additive model (*P* = 0.002, OR = 1.540, 95% CI: 1.160–2.030). Moreover, rs2549782 and rs2287988 also exhibited genotypic differences in the log-additive model with *P* values of 0.003 and 0.002, respectively.

## Discussion

Endoplasmic reticulum aminopeptidases play an important role in shaping the HLA class I peptidome by trimming peptides to the optimal size for MHC-class I-mediated antigen presentation and for preparing the immune system to differentiate between self-derived and foreign antigens. Since the antigen presentation system plays a major role in the interaction between pathogens and the host, ERAPs have been investigated as potential targets and modulators of infectious diseases (Yao et al., 2019). Our previous study indicated that *ERAP1* gene variations are associated with chronic HCV (Liu et al., 2017). In the present study, we analyzed the association of 11 SNPs in *ERAP1* and *ERAP2* genes, and our results, to our knowledge, report for the first time that variations in rs26618, rs2549782, and rs1056893 are associated with TB risk. In addition, rs2549782, rs2248374, rs2287988, and rs1056893 were found to be associated with PTB, EPTB, or re-treated TB.

The ERAP1 roles against virus infection or in autoimmune disease mechanisms through peptide trimming has been investigated in several studies. Aberrant peptide trimming by ERAP1 and a further impaired peptide presentation by HLA-B27 may be involved in the pathogenesis of ankylosing spondylitis (Evans et al., 2011). Comparison of the T cell response between the ERAP1-deficient and wild-type mice infected with the lymphocytic choriomeningitis virus (LCMV) revealed that the magnitude of T cell response to different LCMV epitopes followed an immunodominance hierarchy and was profoundly different between the two groups of mice (York et al., 2006). In chronic HCV infection, the C alleles of rs26618 and the haplotype rs27044-rs30187-rs26618-rs26653 C-C-C-G exhibited the risk association (Liu et al., 2017). In the present study, the C allele of rs26618 in *ERAP1* showed a trend of risk association with TB before Bonferroni correction, and the CC/TT genotype showed a risk factor compared with the role of the CT genotype in the dominant model. In addition, the haplotype of rs27037-rs27044-s30187-rs26618-rs26653-rs3734016-GCCCGC exhibited a risk factor for TB. Several studies have demonstrated that SNPs located at critical structural positions in ERAPs may change the conformation of *ERAP1* and *ERAP2* (Nguyen et al., 2011; Evnouchidou et al., 2012; Hattori and Tsujimoto, 2013). Thus, we deduced that rs26618 may be one of the risk factors for TB infection.

Endoplasmic reticulum aminopeptidases 2 also plays a role in bacterial and viral infections. In 2010, Cagliani et al. studied the ERAPs susceptibility to HIV-1 infection and found that the polymorphisms in *ERAP1* and *ERAP2* genes may have been maintained through long-standing balancing selection, and that ERAP2 conferred resistance to an HIV infection likely via the presentation of a distinctive peptide repertoire to CD8^+^ T cells (Cagliani et al., 2010). The rs2549782, rs2548538, rs2248374, rs2287988, and rs1056893 in *ERAP2* were in high LD, haplotype A (GTAGC) and haplotype B (TAGAT) have been shown to be prevalent in most populations worldwide (Vanhille et al., 2013). Haplotype B (TAGAT) has been associated with a splice-site variant caused by the A allele of rs2248374, resulting in nonsense-mediated RNA decay that precludes protein expression (Vanhille et al., 2013). Besides, the G allele of rs2549782 causes a neoconservative amino acid substitution, resulting in alterations in the ERAP2 enzyme activity and substrate specificity (Evnouchidou et al., 2012). In the present study, all five SNPs in *ERAP2* showed an association with TB in total or in further stratification analysis. The G allele of rs2549782 and the C allele of rs1056893 showed a risk factor for TB infection, and the GT/GG genotype of rs2549782 showed a higher frequency than that of the TT genotype as well as the rs2549782 genotype in the log-additive model in TB patients compared to that in the control. In addition, the higher frequencies of the haplotype A (GTAGC) were investigated between the TB and control groups. In further stratification analysis, the genotypic association was observed in rs2549782 between the PTB and control groups, and in rs2548538 and rs1056893 between the EPTB and control groups. A similar association has also been observed with other infectious diseases such as AIDS or chronic HCV. We found that the A allele of rs2248374 increased the risk for chronic HCV compared with the G allele; Cagliani et al., found that the rs2549782 was associated with HIV in Italians and the TT genotype was over-represented in this HIV-1-exposed seronegative group; Biasin et al., found that rs2549782 may be a risk factor for intravenous drug users exposed to HIV-1 infection in the Spanish population (Cagliani et al., 2010; Biasin et al., 2013; Liu et al., 2017). All these studies suggest that single SNP or SNP haplotypes in *ERAP2* may be associated with TB progression.

One of the major problems associated with TB is its drug resistance. In the present study, to provide insights into TB therapy, we divided the patients with TB into initially treated and re-treated groups according to their treatment classification (China National Health and Family Planning Commission of the People’s Republic of China, 2018a). Interestingly, the minor alleles of all five SNPs in *ERAP2* were found to be the risk factors in the RTB group compared with the control group, and the genotypic differences in the log-additive model of all five SNPs were significant. Furthermore, the differences between the RTB and ITTB groups were investigated in rs2548538 and rs2248374 at the allelic and genotype levels as well as in rs2549782 and rs2287988 at the genotypic level. In addition, the higher frequencies of the haplotype A (GTAGC)in the RTB group than that in the control group suggested the risk of this haplotype. These results indicate that *ERAP2* may play a significant role in TB resistance.

However, in a previous GWAS study in China, no association was identified between TB and *ERAP* genes (Chen et al., 2016a; Zheng et al., 2018). The race, sample size, characteristics of the patients and controls, and the genotyping methods used, are some of the factors that may affect the association study results. The difference in genetic background is an important factor that could affect the association results. For example, rs557011, rs9271378, and rs9272785 in the HLA region were found to be genome-wide significantly associated with TB in the European but not in the Chinese population because these SNPs are rare in the latter (Sveinbjornsson et al., 2016; Zheng et al., 2018). Within the Chinese population, the genetic variants were large, both in Han and other populations. Although the Han Chinese originated from the Huaxia group, the genetic distinction between northern and southern Han has been identified by several genetic markers, such as short tandem repeats (STRs), SNP, and HLA genes (Chu et al., 1998; Su et al., 1999; Shi et al., 2006). Moreover, the integration with other ethnic populations during the past 5000 years and habitation in vast landscapes with an enormous population makes the Han Chinese more complex. In Zhang’s study, they selected three samples from three cities, Shanghai, Nantong, and Shenzhen, all from southern China. In contrast, our samples were selected from the Yunnan Han (three Han generations living in the Yunnan province of Southwestern China), which has been proven to have special genetic characteristics between the southern and northern Han (Shi et al., 2006). Thus, one of the major reasons for the inconsistency between the results of the GWAS study and the present study could be related to the presence of genetic variants in different Han populations. Moreover, the replication cohort should be evaluated in the future. In addition, functional studies will be necessary in order to unravel the mechanism by which ERAP variants affect TB pathogenesis, especially rs2549782, rs2548538, rs2287988, and rs1056983, which have not been studied. Additionally, attempts should also be made to identify other SNPs that induce functional changes, during future research.

## Conclusion

In conclusion, the present study showed an association between the *ERAP1* and *ERAP2* gene polymorphisms and TB in the Han Chinese population. The G allele of rs2549782 and the C allele of rs1056893 in *ERAP2* were found to be the risk factors for TB. In *ERAP1*, the genotype of rs26618, and in *ERAP2*, the genotypes of rs2549782 and rs254978 were associated with TB. Meanwhile, the haplotype rs27037-rs27044-s30187-rs26618-rs26653-rs3734016-GCCCGC in *ERAP1* and rs2549782-rs2248374-rs2287988-rs1056893-GTAGC in *ERAP2* were found to be the risk factors for TB, while the haplotype rs2549782-rs2248374-rs2287988-rs1056893-TAGAT was found to be protective against TB. Moreover, the T allele of rs2548538 and the A allele of rs2248374 may be the risk factors for TB resistance. Further functional studies are required to explore the pathological mechanisms of TB infection.

## Data Availability Statement

The raw data supporting the conclusions of this article will be made available by the authors, without undue reservation.

## Ethics Statement

The studies involving human participants were reviewed and approved by the Institutional Review Board of The Third Hospital of Kunming. The patients/participants provided their written informed consent to participate in this study.

## Author Contributions

LiS and YY conceived and designed the research. SZ, HW, and LB performed the clinical diagnosis and sample collection. SL, NL, LeS, CL, and XZ performed the experiments. SL and NL analyzed the data. LiS wrote the manuscript. All authors have read and approved the final version of the manuscript.

## Conflict of Interest

The authors declare that the research was conducted in the absence of any commercial or financial relationships that could be construed as a potential conflict of interest.
