# Supplementary material for: Polymorphisms in ERAP1 and ERAP2 Genes Are Associated With Tuberculosis in the Han Chinese
Source: Front Genet. 2020 Nov 5;11:566190. doi: 10.3389/fgene.2020.566190 (PMC7676896; doi:10.3389/fgene.2020.566190)

Supplementary Figure 1. The SNaPshot SNP assay group 1 (rs30187, rs26653, rs26618, rs2549782, rs2248374)

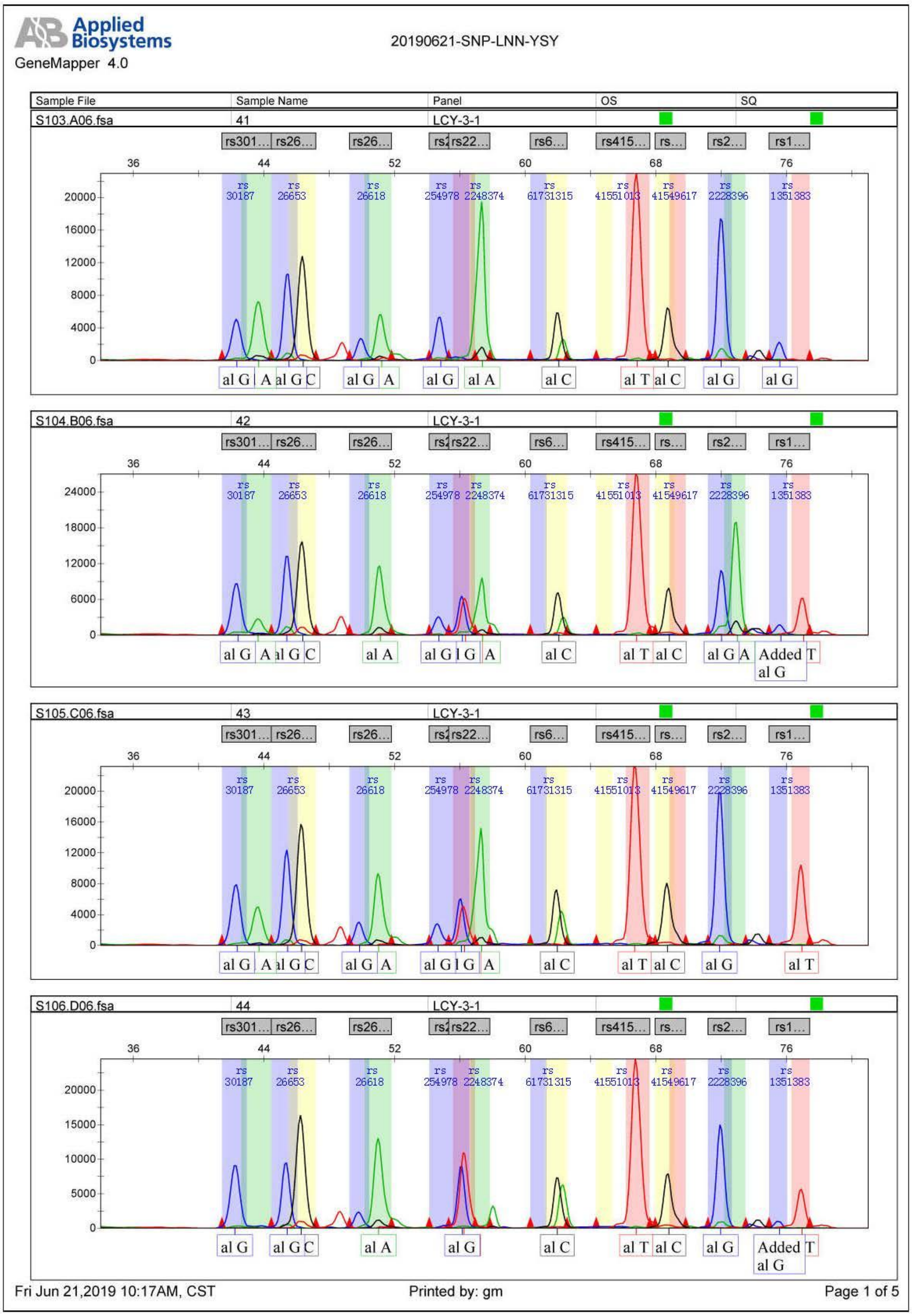

Supplementary Figure 2. The SNaPshot SNP assay group 2 (rs27044)

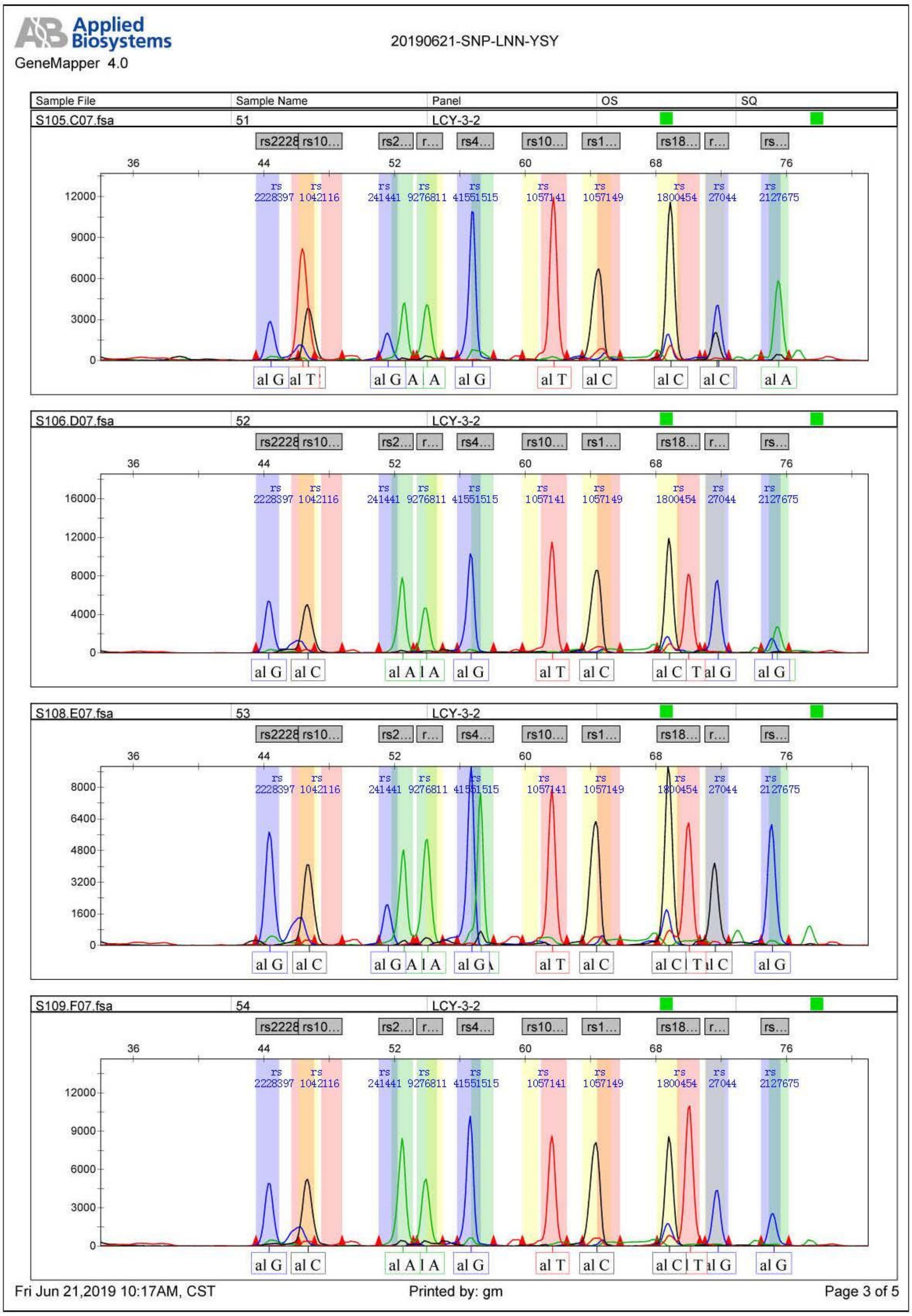

Supplementary Figure 3. The SNaPshot SNP assay group 1 (rs27037, rs3734016, rs2548538, rs2287988, rs1056983)

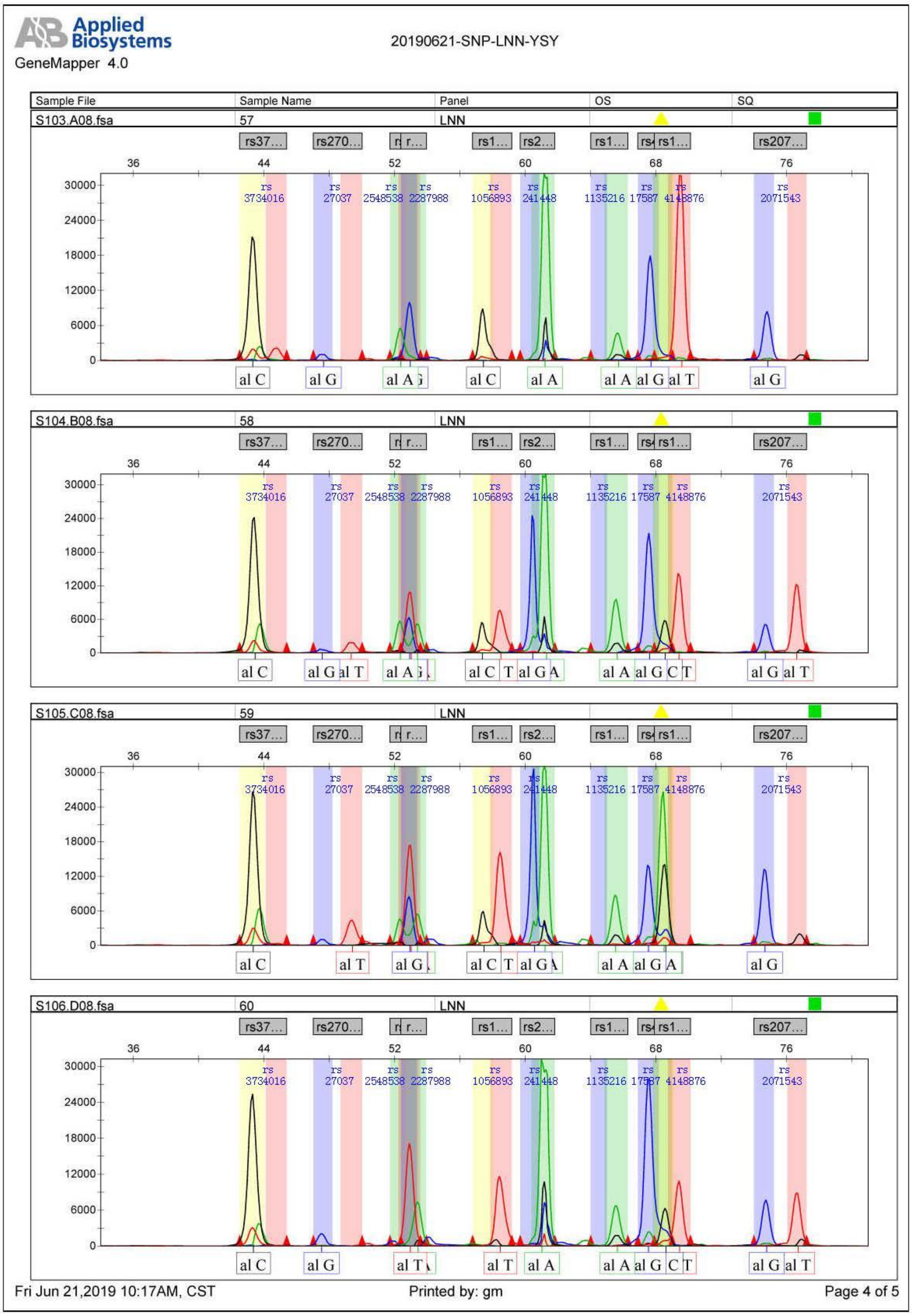

Supplementary Figure 4. The SNP Sequencing map of 11 ERAPs

rs30187:

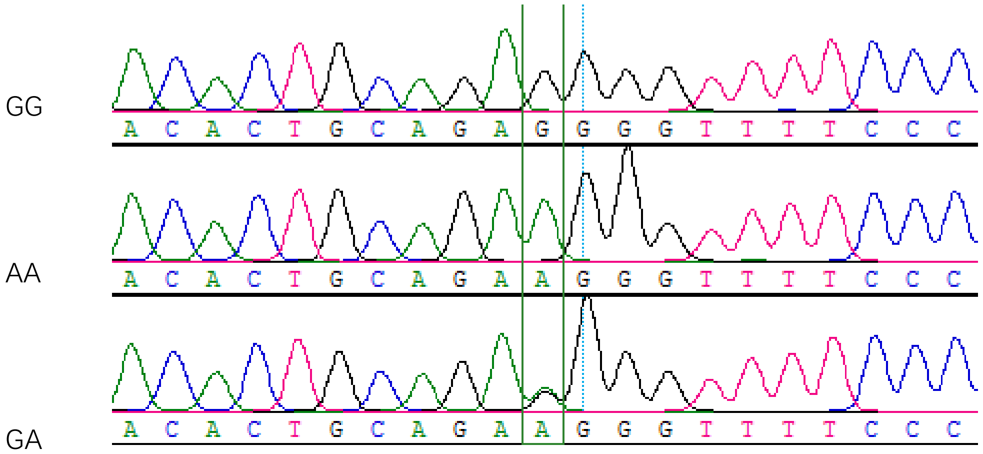

Rs26653:

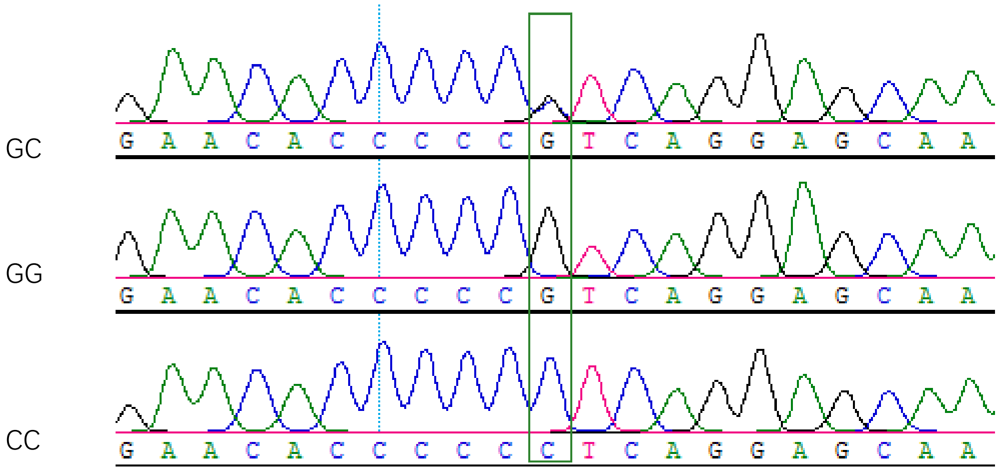

Rs26618:

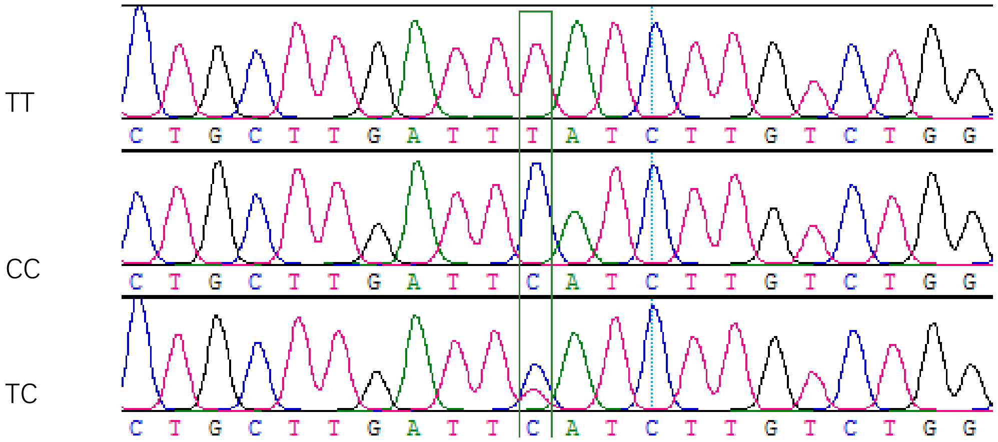

rs2549782:

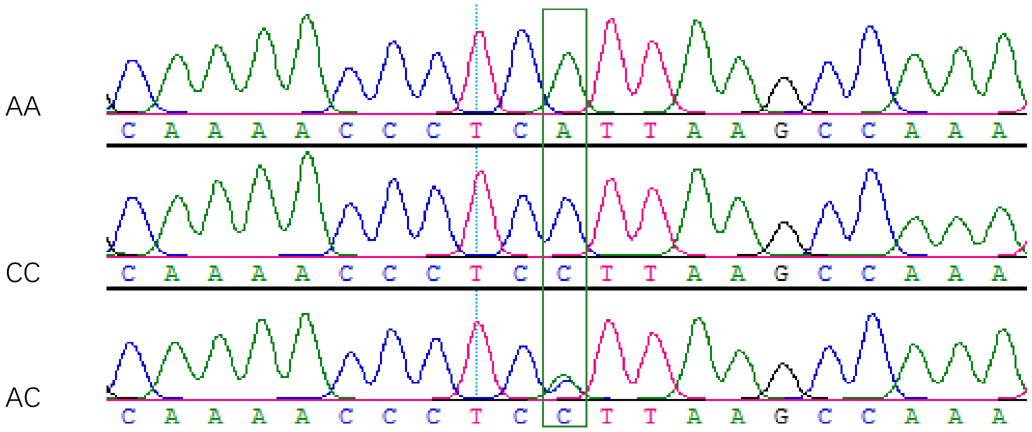

Rs2248374:

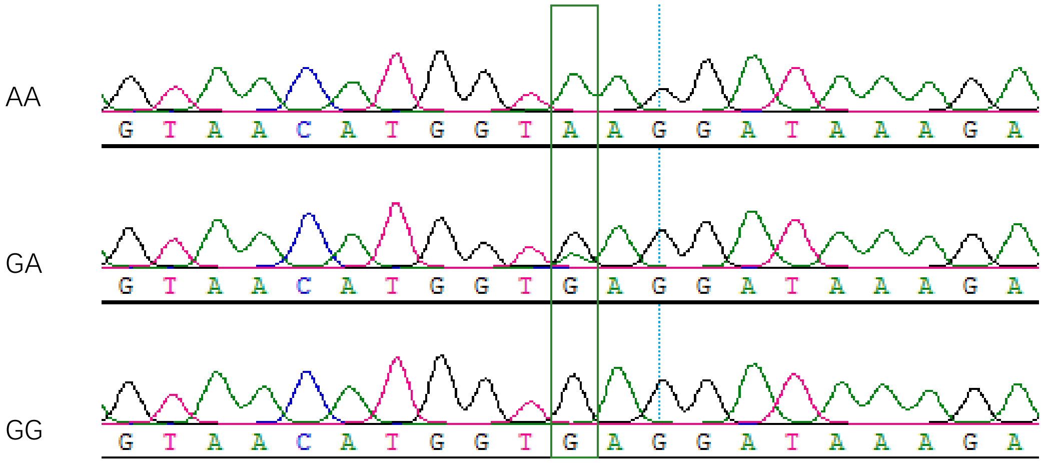

Rs27044:

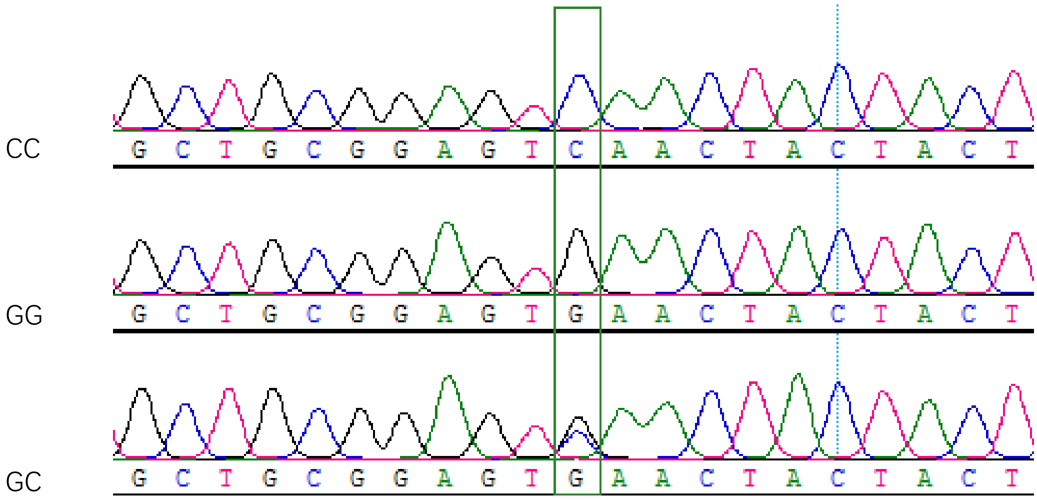

rs3734016

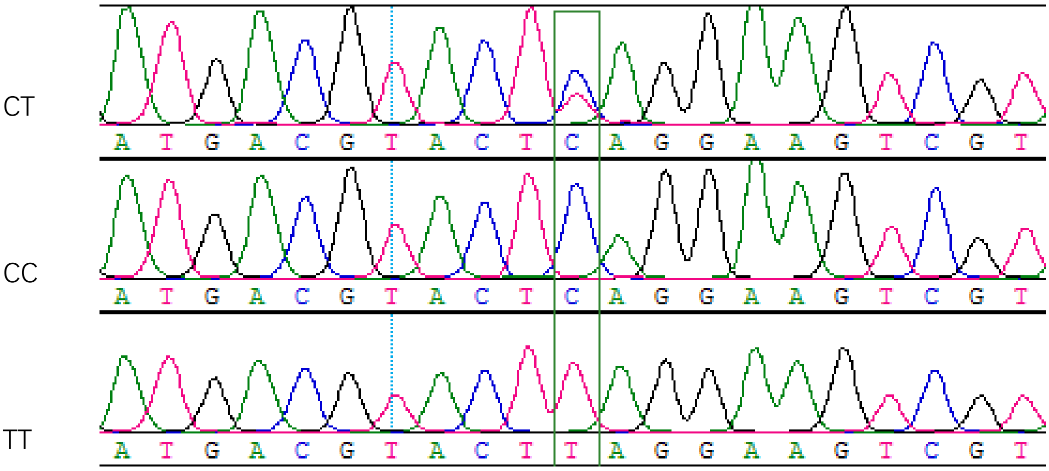

rs27307

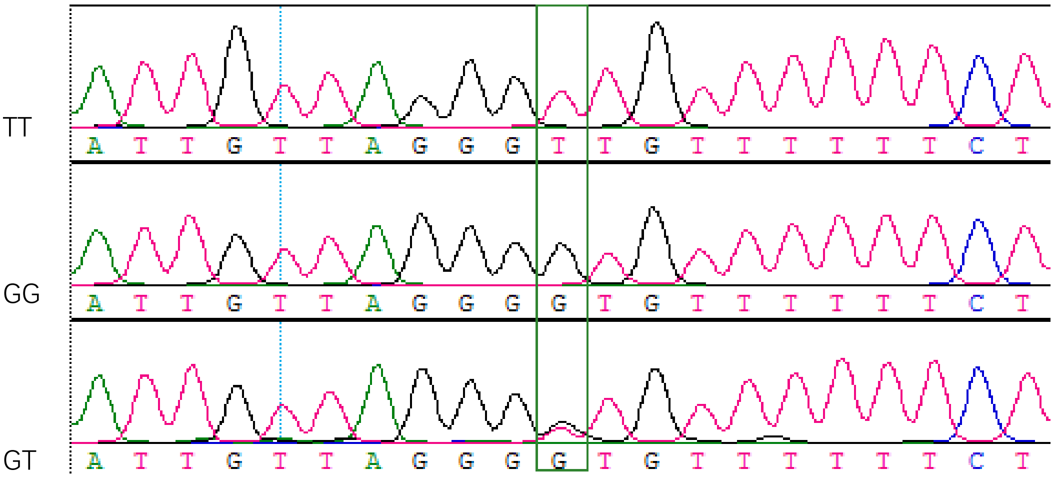

rs2548538:

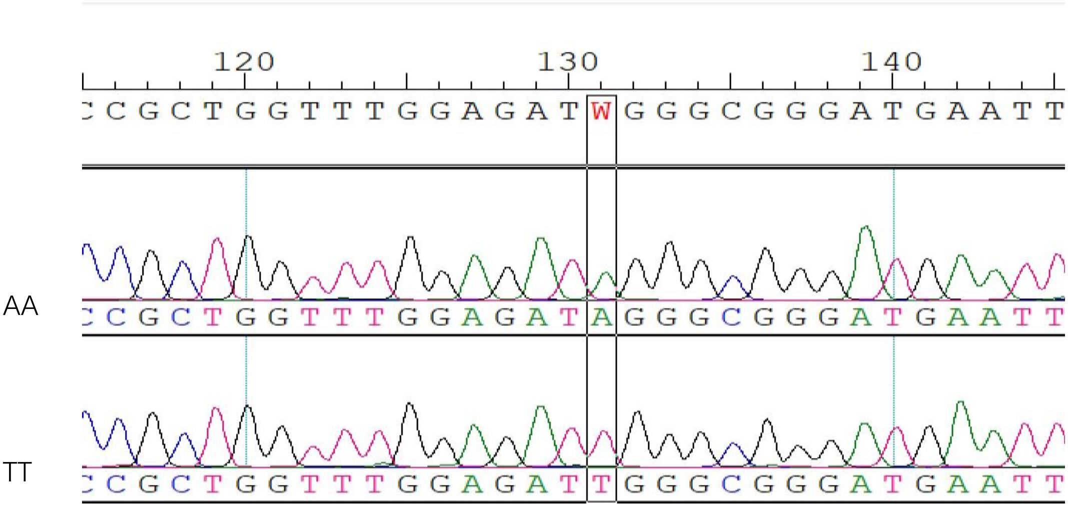

Rs2287988:

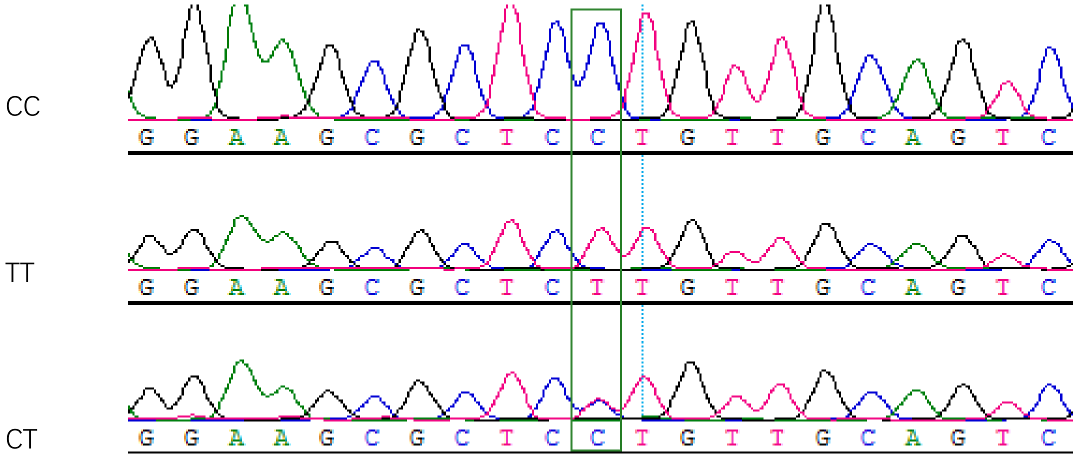

Rs1056893:

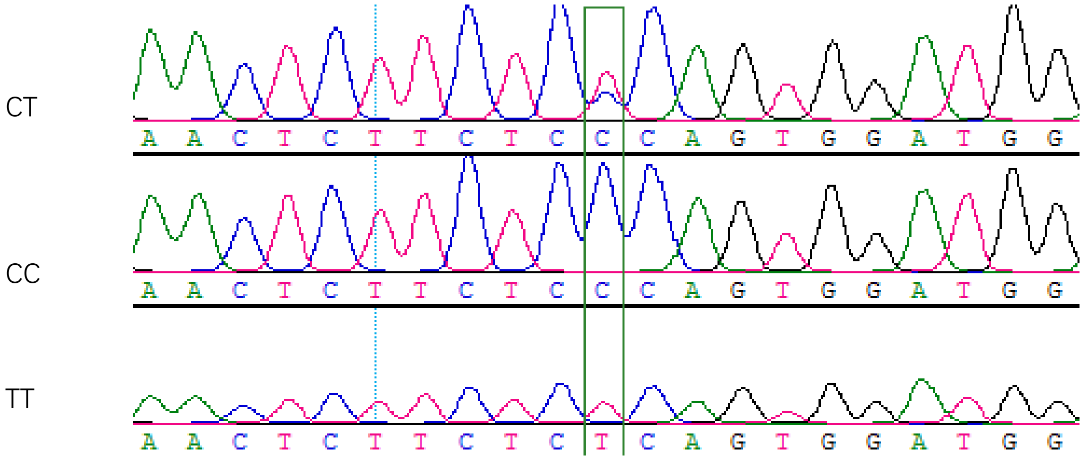

Supplement: Supplementary file 1 [file Data_Sheet_1.PDF]
