# Supplementary material for: Polymorphisms in ERAP1 and ERAP2 Genes Are Associated With Tuberculosis in the Han Chinese
Source: Front Genet. 2020 Nov 5;11:566190. doi: 10.3389/fgene.2020.566190 (PMC7676896; doi:10.3389/fgene.2020.566190)
Supplement: Supplementary file 2 [file Table_1.DOCX]

| **Supplementary table1. Application primers of 11 SNPs in the ERAP1 and ERAP2 genes** |
| --- |
| \| Gene \| Primer name \| Primer Sequence \| Product size(bp) \| \| --- \| --- \| --- \| --- \| \| ERAP1 \| rs27037-F \| TCATCCCGTGACCTTCCTCT \| 307 \| \|  \| rs27037-R \| TGCTACATATCTTTGAAGGGGGC \| \|  \| rs27044-F \| CTGAAATAGCCTTCTGCCCTCT \| 200 \| \|  \| rs27044-R \| ACCTGTGACAATACTGGTCCCT \| \|  \| rs30187-F \| TACATTCCTCCCCCTCACTGT \| 332 \| \|  \| rs30187-R \| ACCTGTCCTCCAGACACGTA \| \|  \| rs26618-F \| TCAACCGCAGGTTTGTCACT \| 320 \| \|  \| rs26618-R \| ATGTGGGGAGAGAAGCCAGT \| \|  \| rs26653-F \| TGACAACTGTGTACGGGAGC \| 226 \| \|  \| rs26653-R \| CTGACCTTCTGGGGAACCAC \| \|  \| rs3734016-F \| GCAGGTGGTGACTATGCAGG \| 304 \| \|  \| rs3734016-R \| AAGATGGTGTTTCTGCCCCTC \| \| ERAP2 \| rs2549782-F \| CTCCTAGTGGTTTGGCAACCT \| 336 \| \|  \| rs2549782-R \| ACAGACTTTGGGGTGACAGATTT \| \|  \| rs2548538-F \| TCTCCCTGTTTAGGATGACTATTTT \| 248 \| \|  \| rs2548538-R \| AAACAAGTAGCAGGAACTGGC \| \|  \| rs2248374-F \| TGTGCACGTTCAGCCAACTA \| 257 \| \|  \| rs2248374-R \| AATCGTGGGGGCTCTCTTTC \| \|  \| rs2287988-F \| AAAGGAATCCCCCTGCTGGT \| 206 \| \|  \| rs2287988-R \| CAAAGTTGCCAGATGAGCTGAG \| \|  \| rs1056893-F \| TGCAGCGTTACCTTCTTCAGT \| 336 \| \|  \| rs1056893-R \| AATGTGAGAGCATGAAACAGGGT \| |

**Supplementary table2. Extension primers of 11 SNPs in the ERAP1 and ERAP2 genes**

| Primer name | Primer sequencing |
| --- | --- |
| rs27037SF | TTTTTTTTTTTTTATCTTTGAATTTCTAAAAGAAAAAACA |
| rs27044SR | TTTTTTTTTTTTTTTTTTTTTTTTTTTTTTTTTTTTTTTTTTTTTTTTTTGAGCGAATGCTGCGGAGT |
| rs30187SR | TTTTTTTTTTTTTTATGAACACTTGGACACTGCAGA |
| rs26618SR | TTTTTTTTTTTTTTTTTTTTTTTTTATGCTGTGCCAGACAAGAT |
| rs26653SR | TTTTTTTTTTTTTTTTTTTTTTAGGTCCTGGAACACCCCC |
| rs3734016SF | TTTTTTTTTTTTCATAATGAACTGGGATGACGTACT |
| rs2549782SF | TTTTTTTTTTTTTTTTTTTTTTTTTTGGTGGAATGATATTTGGCTTAA |
| rs2548538SR | TTTTTTTTTTTTTTTTTTTTTTTTTTTTCCGCTGGTTTGGAGAT |
| rs2248374SF | TTTTTTTTTTTTTTTTTTTTTTTTTTTTTCCCAAGATGACAAGTAACATGGT |
| rs2287988SF | TTTTTTTTTTTTTTTTTTTTTTTTTTTGTGTTCACTCCGACTGCAACA |
| rs1056893SF | TTTTTTTTTTTTTTTTTTTTTTTTTTTTTCAGAAAGCTGCTGAACTCTTCTC |

**Supplementary Table 3. The Hardy-Weinberg equilibrium test for each SNPs in the TB patient and health control groups**

| **Gene** | **SNPs** | **TB** | | **Control** | |
| --- | --- | --- | --- | --- | --- |
|  |  | **χ2** | ***P*** | **χ2** | ***P*** |
| ERAP1 | rs27037 | 1.423 | 0.233 | 0.018 | 0.892 |
|  | rs27044 | 1.303 | 0.254 | 0.600 | 0.439 |
|  | rs30187 | 0.212 | 0.646 | 0.057 | 0.812 |
|  | rs26618 | 3.78 | 0.052 | 0.212 | 0.645 |
|  | rs26653 | 2.813 | 0.094 | 0.396 | 0.529 |
|  | rs3734016 | 2.786 | 0.095 | 0.155 | 0.693 |
| ERAP2 | rs2549782 | 3.772 | 0.052 | 0.021 | 0.886 |
|  | rs2548538 | 0.136 | 0.712 | 0.309 | 0.578 |
|  | rs2248374 | 0.048 | 0.827 | 0.063 | 0.802 |
|  | rs2287988 | 1.112 | 0.292 | 0.232 | 0.630 |
|  | rs1056893 | 0.002 | 0.967 | 0.077 | 0.781 |

**Supplementary table 4. Different Inheritance models analysis of SNPs in ERAP1 and ERAP2 gene between the PTB,EPTB and the control groups**

| **Gene** | **SNPs** | **Model** | **Genotype** | **Control** | **PTB** | **EPTB** |  | **PTB vs Control** | | | |  | **EPTB vs Control*** | | | |
| --- | --- | --- | --- | --- | --- | --- | --- | --- | --- | --- | --- | --- | --- | --- | --- | --- |
|  |  |  |  | **n (freq.)** | **n (freq.)** | **n (freq.)** |  | ***P*** | **OR(95% CI)** | **AIC** | **BIC** |  | ***P*** | **OR(95% CI)** | **AIC** | **BIC** |
| ERAP1 | rs27037 | Codominant | G/G | 149(0.342) | 113(0.349) | 43(0.344) |  |  | Ref | 1041.9 | 1055.8 |  |  | Ref | 573.6 | 595.2 |
|  |  |  | G/T | 210(0.483) | 154(0.475) | 52(0.416) |  | 0.837 | 0.970(0.700-1.330) |  |  |  | 0.51 | 0.850(0.530-1.350) |  |  |
|  |  |  | T/T | 76(0.175) | 57(0.176) | 30(0.240) |  | 0.959 | 0.990(0.650-1.510) |  |  |  | 0.256 | 1.270(0.730-2.230) |  |  |
|  |  | Dominant | G/G | 149(0.342) | 113(0.349) | 43(0.344) |  |  | Ref | 1039.9 | 1049.1 |  |  | Ref | 573.7 | 591 |
|  |  |  | G/T-T/T | 286(0.658) | 211(0.651) | 82(0.656) |  | 0.86 | 0.970(0.720-1.320) |  |  |  | 0.87 | 0.960(0.630-1.480) |  |  |
|  |  | Recessive | G/G-G/T | 359(0.825) | 267(0.824) | 95(0.760) |  |  | Ref | 1039.9 | 1049.2 |  |  | Ref | 572.1 | 589.4 |
|  |  |  | T/T | 76(0.175) | 57(0.176) | 30(0.240) |  | 0.97 | 1.010(0.690-1.470) |  |  |  | 0.19 | 1.400(0.850-2.300) |  |  |
|  |  | Overdominant | G/G-T/T | 225(0.517) | 170(0.525) | 73(0.584) |  |  | Ref | 1039.9 | 1049.1 |  |  | Ref | 572.3 | 589.6 |
|  |  |  | G/T | 210(0.483) | 154(0.475) | 52(0.416) |  | 0.84 | 0.970(0.730-1.290) |  |  |  | 0.23 | 0.770(0.510-1.170) |  |  |
|  |  | Log-additive | --- | --- | --- | --- |  | 0.92 | 0.990(0.810-1.220) | 1039.9 | 1049.2 |  | 0.54 | 1.090(0.820-1.450) | 573.4 | 590.7 |
|  | rs27044 | Codominant | C/C | 116(0.267) | 92(0.284) | 40(0.320) |  |  | Ref | 1041.1 | 1055 |  |  | Ref | 572.9 | 594.6 |
|  |  |  | G/C | 225(0.517) | 170(0.525) | 63(0.504) |  | 0.779 | 0.950(0.680-1.340) |  |  |  | 0.369 | 0.770(0.480-1.230) |  |  |
|  |  |  | G/G | 94(0.216) | 62(0.191) | 22(0.176) |  | 0.391 | 0.830(0.550-1.270) |  |  |  | 0.194 | 0.600(0.330-1.110) |  |  |
|  |  | Dominant | C/C | 116(0.267) | 92(0.284) | 40(0.320) |  |  | Ref | 1039.6 | 1048.9 |  |  | Ref | 571.7 | 589 |
|  |  |  | G/C-G/G | 319(0.733) | 232(0.716) | 85(0.680) |  | 0.6 | 0.920(0.660-1.260) |  |  |  | 0.15 | 0.720(0.460-1.120) |  |  |
|  |  | Recessive | C/C-G/C | 341(0.784) | 262(0.809) | 103(0.824) |  |  | Ref | 1039.2 | 1048.5 |  |  | Ref | 572.1 | 589.5 |
|  |  |  | G/G | 94(0.216) | 62(0.191) | 22(0.176) |  | 0.4 | 0.860(0.600-1.230) |  |  |  | 0.21 | 0.710(0.420-1.210) |  |  |
|  |  | Overdominant | C/C-G/G | 210(0.483) | 154(0.475) | 62(0.496) |  |  | Ref | 1039.9 | 1049.1 |  |  | Ref | 573.7 | 591 |
|  |  |  | G/C | 225(0.517) | 170(0.525) | 63(0.504) |  | 0.84 | 1.030(0.770-1.370) |  |  |  | 0.77 | 0.940(0.620-1.420) |  |  |
|  |  | Log-additive | --- | --- | --- | --- |  | 0.41 | 0.920(0.740-1.130) | 1039.2 | 1048.5 |  | 0.094 | 0.780(0.570-1.050) | 570.9 | 588.3 |
|  | rs30187 | Codominant | C/C | 108(0.248) | 91(0.281) | 36(0.288) |  |  | Ref | 1040.6 | 1054.5 |  |  | Ref | 573.7 | 595.4 |
|  |  |  | C/T | 215(0.494) | 158(0.488) | 62(0.496) |  | 0.439 | 0.870(0.620-1.230) |  |  |  | 0.547 | 0.830(0.510-1.340) |  |  |
|  |  |  | T/T | 112(0.258) | 75(0.231) | 27(0.216) |  | 0.265 | 0.790(0.530-1.190) |  |  |  | 0.26 | 0.660(0.370-1.180) |  |  |
|  |  | Dominant | C/C | 108(0.248) | 91(0.281) | 36(0.288) |  |  | Ref | 1038.9 | 1048.2 |  |  | Ref | 572.5 | 589.8 |
|  |  |  | C/T-T/T | 327(0.752) | 233(0.719) | 89(0.712) |  | 0.31 | 0.850(0.610-1.170) |  |  |  | 0.26 | 0.770(0.490-1.210) |  |  |
|  |  | Recessive | C/C-C/T | 323(0.742) | 249(0.768) | 98(0.784) |  |  | Ref | 1039.2 | 1048.5 |  |  | Ref | 572.3 | 589.6 |
|  |  |  | T/T | 112(0.258) | 75(0.231) | 27(0.216) |  | 0.41 | 0.870(0.620-1.220) |  |  |  | 0.23 | 0.740(0.450-1.220) |  |  |
|  |  | Overdominant | C/C-T/T | 220(0.506) | 166(0.512) | 63(0.504) |  |  | Ref | 1039.9 | 1049.1 |  |  | Ref | 573.8 | 591.1 |
|  |  |  | C/T | 215(0.494) | 158(0.488) | 62(0.496) |  | 0.86 | 0.970(0.730-1.300) |  |  |  | 0.97 | 1.010(0.670-1.520) |  |  |
|  |  | Log-additive | --- | --- | --- | --- |  | 0.26 | 0.890(0.730-1.090) | 1038.7 | 1047.9 |  | 0.16 | 0.810(0.610-1.080) | 571.7 | 589.1 |
|  | rs26618 | Codominant | T/T | 238(0.547) | 145(0.448) | 55(0.440) |  |  | Ref | 1034.4 | 1048.3 |  |  | Ref | 570.2 | 591.9 |
|  |  |  | C/T | 165(0.379) | 152(0.469) | 61(0.488) |  | 0.007 | 1.510(1.120-2.050) |  |  |  | 0.026 | 1.670(1.090-2.560) |  |  |
|  |  |  | C/C | 32(0.074) | 27(0.083) | 9(0.072) |  | 0.246 | 1.380(0.800-2.410) |  |  |  | 0.628 | 1.230(0.550-2.780) |  |  |
|  |  | Dominant | T/T | 238(0.547) | 145(0.448) | 55(0.440) |  |  | Ref | 1032.5 | 1041.8 |  |  | Ref | 568.8 | 586.1 |
|  |  |  | C/T-C/C | 197(0.453) | 179(0.552) | 70(0.560) |  | 0.0066 | 1.490(1.120-1.990) |  |  |  | 0.026 | 1.600(1.060-2.410) |  |  |
|  |  | Recessive | T/T-C/T | 403(0.926) | 297(0.917) | 116(0.928) |  |  | Ref | 1039.7 | 1048.9 |  |  | Ref | 573.7 | 591.1 |
|  |  |  | C/C | 32(0.074) | 27(0.083) | 9(0.072) |  | 0.62 | 1.140(0.670-1.950) |  |  |  | 0.94 | 0.970(0.440-2.120) |  |  |
|  |  | Overdominant | T/T-C/C | 270(0.621) | 172(0.531) | 64(0.512) |  |  | Ref | 1033.8 | 1043 |  |  | Ref | 568.5 | 585.8 |
|  |  |  | C/T | 165(0.379) | 152(0.469) | 61(0.488) |  | 0.013 | 1.450(1.080-1.940) |  |  |  | 0.022 | 1.620(1.070-2.460) |  |  |
|  |  | Log-additive | --- | --- | --- | --- |  | 0.019 | 1.310(1.050-1.650) | 1034.4 | 1043.6 |  | 0.085 | 1.320(0.960-1.820) | 570.8 | 588.1 |
|  | rs26653 | Codominant | C/C | 127(0.292) | 82(0.253) | 28(0.224) |  |  | Ref | 1038.9 | 1052.8 |  |  | Ref | 572.6 | 594.3 |
|  |  |  | G/C | 210(0.483) | 177(0.546) | 66(0.528) |  | 0.127 | 1.310(0.930-1.840) |  |  |  | 0.158 | 1.530(0.920-2.560) |  |  |
|  |  |  | G/G | 98(0.225) | 65(0.201) | 31(0.248) |  | 0.9 | 1.030(0.680-1.560) |  |  |  | 0.217 | 1.540(0.850-2.780) |  |  |
|  |  | Dominant | C/C | 127(0.292) | 82(0.253) | 28(0.224) |  |  | Ref | 1038.5 | 1047.8 |  |  | Ref | 570.6 | 587.9 |
|  |  |  | G/C-G/G | 308(0.708) | 242(0.747) | 97(0.776) |  | 0.23 | 1.220(0.880-1.680) |  |  |  | 0.077 | 1.530(0.950-2.490) |  |  |
|  |  | Recessive | C/C-G/C | 337(0.775) | 259(0.799) | 94(0.752) |  |  | Ref | 1039.2 | 1048.5 |  |  | Ref | 573.4 | 590.7 |
|  |  |  | G/G | 98(0.225) | 65(0.201) | 31(0.248) |  | 0.41 | 0.860(0.610-1.230) |  |  |  | 0.54 | 1.160(0.720-1.870) |  |  |
|  |  | Overdominant | C/C-G/G | 225(0.517) | 147(0.454) | 59(0.472) |  |  | Ref | 1036.9 | 1046.2 |  |  | Ref | 572.6 | 590 |
|  |  |  | G/C | 210(0.483) | 177(0.546) | 66(0.528) |  | 0.083 | 1.290(0.970-1.720) |  |  |  | 0.29 | 1.250(0.830-1.880) |  |  |
|  |  | Log-additive | --- | --- | --- | --- |  | 0.78 | 1.030(0.840-1.270) | 1039.8 | 1049.1 |  | 0.15 | 1.240(0.930-1.650) | 571.6 | 589 |
|  | rs3734016 | Codominant | C/C | 306(0.703) | 251(0.775) | 96(0.768) |  |  | Ref | 1035.9 | 1049.8 |  |  | Ref | 573.7 | 595.3 |
|  |  |  | C/T | 119(0.274) | 64(0.198) | 27(0.216) |  | 0.017 | 0.660(0.460-0.930) |  |  |  | 0.181 | 0.700(0.430-1.150) |  |  |
|  |  |  | T/T | 10(0.023) | 9(0.028) | 2(0.016) |  | 0.843 | 1.100(0.440-2.740) |  |  |  | 0.562 | 1.080(0.230-5.180) |  |  |
|  |  | Dominant | C/C | 306(0.703) | 251(0.775) | 96(0.768) |  |  | Ref | 1035 | 1044.3 |  |  | Ref | 571.9 | 589.2 |
|  |  |  | C/T-T/T | 129(0.297) | 73(0.225) | 29(0.232) |  | 0.027 | 0.690(0.490-0.960) |  |  |  | 0.18 | 0.720(0.450-1.160) |  |  |
|  |  | Recessive | C/C-C/T | 425(0.977) | 315(0.972) | 123(0.984) |  |  | Ref | 1039.7 | 1049 |  |  | Ref | 573.7 | 591 |
|  |  |  | T/T | 10(0.023) | 9(0.028) | 2(0.016) |  | 0.68 | 1.210(0.490-3.020) |  |  |  | 0.84 | 1.180(0.250-5.620) |  |  |
|  |  | Overdominant | C/C-T/T | 316(0.726) | 260(0.802) | 98(0.784) |  |  | Ref | 1034 | 1043.2 |  |  | Ref | 571.7 | 589 |
|  |  |  | C/T | 119(0.274) | 64(0.198) | 27(0.216) |  | 0.015 | 0.650(0.460-0.920) |  |  |  | 0.15 | 0.700(0.430-1.150) |  |  |
|  |  | Log-additive | --- | --- | --- | --- |  | 0.072 | 0.770(0.570-1.030) | 1036.7 | 1045.9 |  | 0.23 | 0.770(0.500-1.190) | 572.3 | 589.7 |
| ERAP2 | rs2549782 | Codominant | T/T | 147(0.338) | 72(0.222) | 34(0.272) |  |  | Ref | 1029.5 | 1043.4 |  |  | Ref | 572.4 | 594 |
|  |  |  | G/T | 213(0.490) | 184(0.568) | 61(0.488) |  | 0.001 | 1.760(1.250-2.490) |  |  |  | 0.372 | 1.270(0.780-2.050) |  |  |
|  |  |  | G/G | 75(0.172) | 68(0.210) | 30(0.240) |  | 0.005 | 1.850(1.200-2.850) |  |  |  | 0.056 | 1.730(0.970-3.080) |  |  |
|  |  | Dominant | T/T | 147(0.338) | 72(0.222) | 34(0.272) |  |  | Ref | 1027.6 | 1036.8 |  |  | Ref | 571.7 | 589 |
|  |  |  | G/T-G/G | 288(0.662) | 252(0.778) | 91(0.728) |  | 0.0004 | 1.790(1.290-2.480) |  |  |  | 0.15 | 1.390(0.880-2.180) |  |  |
|  |  | Recessive | T/T-G/T | 360(0.828) | 256(0.790) | 95(0.760) |  |  | Ref | 1038.2 | 1047.5 |  |  | Ref | 571.3 | 588.6 |
|  |  |  | G/G | 75(0.172) | 68(0.210) | 30(0.240) |  | 0.19 | 1.280(0.880-1.840) |  |  |  | 0.12 | 1.490(0.910-2.450) |  |  |
|  |  | Overdominant | T/T-G/G | 222(0.510) | 140(0.432) | 64(0.512) |  |  | Ref | 1035.3 | 1044.6 |  |  | Ref | 573.7 | 591.1 |
|  |  |  | G/T | 213(0.490) | 184(0.568) | 61(0.488) |  | 0.033 | 1.370(1.030-1.830) |  |  |  | 0.93 | 1.020(0.680-1.530) |  |  |
|  |  | Log-additive | --- | --- | --- | --- |  | 0.0022 | 1.390(1.120-1.720) | 1030.5 | 1039.8 |  | 0.067 | 1.310(0.980-1.750) | 570.4 | 587.7 |
|  | rs2548538 | Codominant | A/A | 142(0.326) | 95(0.293) | 25(0.200) |  |  | Ref | 1040.1 | 1054 |  |  | Ref | 564.8 | 586.5 |
|  |  |  | A/T | 218(0.501) | 162(0.500) | 66(0.528) |  | 0.532 | 1.110(0.800-1.540) |  |  |  | 0.035 | 1.760(1.050-2.960) |  |  |
|  |  |  | T/T | 75(0.172) | 67(0.207) | 34(0.272) |  | 0.176 | 1.340(0.880-2.030) |  |  |  | 0.001 | 2.710(1.480-4.970) |  |  |
|  |  | Dominant | A/A | 142(0.326) | 95(0.293) | 25(0.200) |  |  | Ref | 1038.9 | 1048.2 |  |  | Ref | 565.5 | 582.8 |
|  |  |  | A/T-T/T | 293(0.674) | 229(0.707) | 100(0.800) |  | 0.33 | 1.170(0.860-1.600) |  |  |  | 0.004 | 2.000(1.220-3.280) |  |  |
|  |  | Recessive | A/A-A/T | 360(0.828) | 257(0.793) | 91(0.728) |  |  | Ref | 1038.5 | 1047.7 |  |  | Ref | 567.7 | 585 |
|  |  |  | T/T | 75(0.172) | 67(0.207) | 34(0.272) |  | 0.23 | 1.250(0.870-1.800) |  |  |  | 0.014 | 1.860(1.150-3.020) |  |  |
|  |  | Overdominant | A/A-T/T | 217(0.499) | 162(0.500) | 59(0.472) |  |  | Ref | 1039.9 | 1049.2 |  |  | Ref | 573.4 | 590.7 |
|  |  |  | A/T | 218(0.501) | 162(0.500) | 66(0.528) |  | 0.98 | 1.000(0.750-1.330) |  |  |  | 0.57 | 1.130(0.750-1.700) |  |  |
|  |  | Log-additive | --- | --- | --- | --- |  | 0.19 | 1.150(0.930-1.410) | 1038.2 | 1047.4 |  | 0.001 | 1.650(1.220-2.220) | 562.9 | 580.2 |
|  | rs2248374 | Codominant | G/G | 149(0.342) | 92(0.284) | 33(0.264) |  |  | Ref | 1038.6 | 1052.5 |  |  | Ref | 571.9 | 593.5 |
|  |  |  | G/A | 209(0.480) | 164(0.506) | 62(0.496) |  | 0.155 | 1.270(0.910-1.770) |  |  |  | 0.224 | 1.370(0.850-2.230) |  |  |
|  |  |  | A/A | 77(0.177) | 68(0.210) | 30(0.240) |  | 0.092 | 1.430(0.940-2.170) |  |  |  | 0.049 | 1.780(0.990-3.180) |  |  |
|  |  | Dominant | G/G | 149(0.342) | 92(0.284) | 33(0.264) |  |  | Ref | 1036.9 | 1046.2 |  |  | Ref | 570.8 | 588.1 |
|  |  |  | G/A-A/A | 286(0.658) | 232(0.716) | 92(0.736) |  | 0.086 | 1.310(0.960-1.800) |  |  |  | 0.085 | 1.480(0.940-2.340) |  |  |
|  |  | Recessive | G/G-G/A | 358(0.823) | 256(0.790) | 95(0.760) |  |  | Ref | 1038.6 | 1047.9 |  |  | Ref | 571.6 | 588.9 |
|  |  |  | A/A | 77(0.177) | 68(0.210) | 30(0.240) |  | 0.26 | 1.230(0.860-1.780) |  |  |  | 0.14 | 1.460(0.890-2.400) |  |  |
|  |  | Overdominant | G/G-A/A | 226(0.520) | 160(0.494) | 63(0.504) |  |  | Ref | 1039.4 | 1048.7 |  |  | Ref | 573.6 | 590.9 |
|  |  |  | G/A | 209(0.480) | 164(0.506) | 62(0.496) |  | 0.48 | 1.110(0.830-1.480) |  |  |  | 0.68 | 1.090(0.720-1.640) |  |  |
|  |  | Log-additive | --- | --- | --- | --- |  | 0.076 | 1.200(0.980-1.480) | 1036.8 | 1046 |  | 0.049 | 1.330(1.000-1.780) | 569.9 | 587.2 |
|  | rs2287988 | Codominant | A/A | 150(0.345) | 86(0.265) | 33(0.264) |  |  | Ref | 1035.9 | 1049.8 |  |  | Ref | 570.7 | 592.3 |
|  |  |  | G/A | 215(0.494) | 173(0.534) | 62(0.496) |  | 0.045 | 1.400(1.010-1.960) |  |  |  | 0.259 | 1.320(0.820-2.140) |  |  |
|  |  |  | G/G | 70(0.161) | 65(0.201) | 30(0.240) |  | 0.027 | 1.620(1.050-2.490) |  |  |  | 0.021 | 1.970(1.090-3.540) |  |  |
|  |  | Dominant | A/A | 150(0.345) | 86(0.265) | 33(0.264) |  |  | Ref | 1034.4 | 1043.7 |  |  | Ref | 570.8 | 588.1 |
|  |  |  | G/A-G/G | 285(0.655) | 238(0.735) | 92(0.736) |  | 0.019 | 1.460(1.060-2.000) |  |  |  | 0.085 | 1.480(0.940-2.340) |  |  |
|  |  | Recessive | A/A-G/A | 365(0.839) | 259(0.799) | 95(0.760) |  |  | Ref | 1037.9 | 1047.2 |  |  | Ref | 570 | 587.3 |
|  |  |  | G/G | 70(0.161) | 65(0.201) | 30(0.240) |  | 0.16 | 1.310(0.900-1.900) |  |  |  | 0.052 | 1.660(1.000-2.730) |  |  |
|  |  | Overdominant | A/A-G/G | 220(0.506) | 151(0.466) | 63(0.504) |  |  | Ref | 1038.7 | 1048 |  |  | Ref | 573.7 | 591.1 |
|  |  |  | G/A | 215(0.494) | 173(0.534) | 62(0.496) |  | 0.28 | 1.170(0.880-1.560) |  |  |  | 0.94 | 1.010(0.670-1.530) |  |  |
|  |  | Log-additive | --- | --- | --- | --- |  | 0.018 | 1.290(1.040-1.590) | 1034.3 | 1043.6 |  | 0.025 | 1.400(1.040-1.880) | 568.7 | 586.1 |
|  | rs1056893 | Codominant | T/T | 161(0.370) | 95(0.293) | 32(0.256) |  |  | Ref | 1035.9 | 1049.8 |  |  | Ref | 567.7 | 589.4 |
|  |  |  | C/T | 205(0.471) | 162(0.500) | 62(0.496) |  | 0.079 | 1.340(0.970-1.860) |  |  |  | 0.081 | 1.550(0.950-2.520) |  |  |
|  |  |  | C/C | 69(0.159) | 67(0.207) | 31(0.248) |  | 0.02 | 1.650(1.080-2.510) |  |  |  | 0.004 | 2.300(1.280-4.130) |  |  |
|  |  | Dominant | T/T | 161(0.370) | 95(0.293) | 32(0.256) |  |  | Ref | 1035 | 1044.2 |  |  | Ref | 567.8 | 585.1 |
|  |  |  | C/T-C/C | 274(0.630) | 229(0.707) | 93(0.744) |  | 0.026 | 1.420(1.040-1.930) |  |  |  | 0.015 | 1.740(1.100-2.760) |  |  |
|  |  | Recessive | T/T-C/T | 366(0.841) | 257(0.793) | 94(0.752) |  |  | Ref | 1037 | 1046.3 |  |  | Ref | 568.9 | 586.2 |
|  |  |  | C/C | 69(0.159) | 67(0.207) | 31(0.248) |  | 0.088 | 1.380(0.950-2.010) |  |  |  | 0.028 | 1.770(1.080-2.910) |  |  |
|  |  | Overdominant | T/T-C/C | 230(0.529) | 162(0.500) | 63(0.504) |  |  | Ref | 1039.3 | 1048.6 |  |  | Ref | 573.5 | 590.8 |
|  |  |  | C/T | 205(0.471) | 162(0.500) | 62(0.496) |  | 0.43 | 1.120(0.840-1.500) |  |  |  | 0.58 | 1.120(0.740-1.700) |  |  |
|  |  | Log-additive | --- | --- | --- | --- |  | 0.015 | 1.290(1.050-1.590) | 1034 | 1043.3 |  | 0.005 | 1.520(1.140-2.030) | 565.7 | 583 |

Note: The statistical significant threshold was set at P<0.0045 after Bonferroni correction

*The P-value, OR and 95%CIs of pairs comparison between PTB and control and EPTB and control were calculated on the basis of the logistic regression model adjusted by age

**Supplementary table 5. Different Inheritance models analysis of SNPs in ERAP1 and ERAP2 gene between the ITTB, RTB and the control groups**

| **SNPs** | **Model** | **genotype** | **control** | **ITTB** | **RTB** | **ITTB vs Control*** | | | | **RTB vs Control** | | | | **RTB vs ITTB**** | | | |
| --- | --- | --- | --- | --- | --- | --- | --- | --- | --- | --- | --- | --- | --- | --- | --- | --- | --- |
|  |  |  | **n (freq.)** | **n (freq.)** | **n (freq.)** | ***P*** | **OR(95% CI)** | **AIC** | **BIC** | ***P*** | **OR(95% CI)** | **AIC** | **BIC** | ***P*** | **OR(95% CI)** | **AIC** | **BIC** |
| rs27037 | Codominant | G/G | 149(0.342) | 89(0.320) | 66(0.386) |  | 1 | 956.5 | 979.4 |  | Ref | 724.9 | 738.1 |  | Ref | 599 | 619.5 |
|  |  | G/T | 210(0.483) | 136(0.489) | 71(0.415) | 0.641 | 1.120(0.800-1.590) |  |  | 0.18 | 0.760(0.510-1.130) |  |  | 0.108 | 0.690(0.450-1.060) |  |  |
|  |  | T/T | 76(0.175) | 53(0.191) | 34(0.199) | 0.488 | 1.160(0.750-1.800) |  |  | 0.969 | 1.010(0.610-1.660) |  |  | 0.596 | 0.840(0.490-1.440) |  |  |
|  | Dominant | G/G | 149(0.342) | 89(0.320) | 66(0.386) |  | 1 | 954.5 | 972.8 |  | Ref | 724.1 | 732.9 |  | Ref | 597.6 | 614 |
|  |  | G/T-T/T | 286(0.658) | 189(0.680) | 105(0.614) | 0.45 | 1.130(0.820-1.570) |  |  | 0.32 | 0.830(0.570-1.200) |  |  | 0.13 | 0.730(0.490-1.090) |  |  |
|  | Recessive | G/G-G/T | 359(0.825) | 225(0.809) | 137(0.801) |  | 1 | 955 | 973.2 |  | Ref | 724.7 | 733.5 |  | Ref | 599.9 | 616.3 |
|  |  | T/T | 76(0.175) | 53(0.191) | 34(0.199) | 0.69 | 1.080(0.730-1.600) |  |  | 0.49 | 1.170(0.750-1.840) |  |  | 0.88 | 1.040(0.640-1.680) |  |  |
|  | Overdominant | G/G-T/T | 225(0.517) | 142(0.511) | 100(0.585) |  | 1 | 955 | 973.2 |  | Ref | 722.9 | 731.7 |  | Ref | 597.4 | 613.8 |
|  |  | G/T | 210(0.483) | 136(0.489) | 71(0.415) | 0.68 | 1.070(0.790-1.450) |  |  | 0.13 | 0.760(0.530-1.090) |  |  | 0.11 | 0.730(0.500-1.080) |  |  |
|  | Log-additive | --- | --- | --- | --- | 0.47 | 1.080(0.870-1.340) | 954.6 | 972.9 | 0.76 | 0.960(0.750-1.230) | 725 | 733.9 | 0.35 | 0.880(0.670-1.15) | 599 | 615.5 |
| rs27044 | Codominant | C/C | 116(0.267) | 75(0.270) | 56(0.328) |  | 1 | 953.2 | 976 |  | Ref | 724.2 | 737.4 |  | Ref | 594.9 | 615.5 |
|  |  | G/C | 225(0.517) | 158(0.568) | 76(0.444) | 0.648 | 1.110(0.770-1.580) |  |  | 0.088 | 0.700(0.460-1.060) |  |  | 0.05 | 0.630(0.410-0.990) |  |  |
|  |  | G/G | 94(0.216) | 45(0.162) | 39(0.228) | 0.199 | 0.730(0.460-1.160) |  |  | 0.545 | 0.860(0.530-1.400) |  |  | 0.596 | 1.140(0.660-1.990) |  |  |
|  | Dominant | C/C | 116(0.267) | 75(0.270) | 56(0.328) |  | 1 | 955.1 | 973.4 |  | Ref | 722.9 | 731.8 |  | Ref | 598 | 614.4 |
|  |  | G/C-G/G | 319(0.733) | 203(0.730) | 115(0.672) | 0.97 | 0.990(0.710-1.400) |  |  | 0.14 | 0.750(0.510-1.100) |  |  | 0.17 | 0.750(0.490-1.130) |  |  |
|  | Recessive | C/C-G/C | 341(0.784) | 233(0.838) | 132(0.772) |  | 1 | 951.5 | 969.8 |  | Ref | 725 | 733.9 |  | Ref | 597 | 613.4 |
|  |  | G/G | 94(0.216) | 45(0.162) | 39(0.228) | 0.056 | 0.680(0.460-1.020) |  |  | 0.75 | 1.070(0.700-1.640) |  |  | 0.09 | 1.520(0.940-2.460) |  |  |
|  | Overdominant | C/C-G/G | 210(0.483) | 120(0.432) | 95(0.556) |  | 1 | 952.9 | 971.2 |  | Ref | 722.5 | 731.3 |  | Ref | 593.2 | 609.6 |
|  |  | G/C | 225(0.517) | 158(0.568) | 76(0.444) | 0.14 | 1.260(0.930-1.710) |  |  | 0.11 | 0.750(0.520-1.070) |  |  | 0.0095 | 0.600(0.410-0.880) |  |  |
|  | Log-additive | --- | --- | --- | --- | 0.26 | 0.880(0.700-1.100) | 953.8 | 972.1 | 0.44 | 0.910(0.710-1.170) | 724.6 | 733.4 | 0.95 | 1.010(0.760-1.330) | 599.9 | 616.3 |
| rs30187 | Codominant | C/C | 108(0.248) | 72(0.259) | 55(0.322) |  | 1 | 955.8 | 978.6 |  | Ref | 723.7 | 736.9 |  | Ref | 598 | 618.5 |
|  |  | C/T | 215(0.494) | 145(0.522) | 74(0.433) | 0.951 | 1.020(0.700-1.470) |  |  | 0.066 | 0.680(0.440-1.030) |  |  | 0.078 | 0.650(0.410-1.020) |  |  |
|  |  | T/T | 112(0.258) | 61(0.219) | 42(0.246) | 0.358 | 0.820(0.530-1.260) |  |  | 0.212 | 0.740(0.460-1.190) |  |  | 0.699 | 0.890(0.520-1.510) |  |  |
|  | Dominant | C/C | 108(0.248) | 72(0.259) | 55(0.322) |  | 1 | 955 | 973.3 |  | Ref | 721.9 | 730.7 |  | Ref | 597.6 | 614 |
|  |  | C/T-T/T | 327(0.752) | 206(0.741) | 116(0.678) | 0.77 | 0.950(0.670-1.340) |  |  | 0.07 | 0.700(0.470-1.030) |  |  | 0.13 | 0.720(0.470-1.100) |  |  |
|  | Recessive | C/C-C/T | 323(0.742) | 217(0.781) | 129(0.754) |  | 1 | 953.8 | 972.1 |  | Ref | 725 | 733.9 |  | Ref | 599.5 | 615.9 |
|  |  | T/T | 112(0.258) | 61(0.219) | 42(0.246) | 0.25 | 0.810(0.570-1.160) |  |  | 0.76 | 0.940(0.620-1.410) |  |  | 0.51 | 1.160(0.740-1.830) |  |  |
|  | Overdominant | C/C-T/T | 220(0.506) | 133(0.478) | 97(0.567) |  | 1 | 954.6 | 972.9 |  | Ref | 723.3 | 732.1 |  | Ref | 596.2 | 612.6 |
|  |  | C/T | 215(0.494) | 145(0.522) | 74(0.433) | 0.46 | 1.120(0.830-1.520) |  |  | 0.17 | 0.780(0.550-1.110) |  |  | 0.054 | 0.690(0.470-1.010) |  |  |
|  | Log-additive | --- | --- | --- | --- | 0.37 | 0.910(0.730-1.130) | 954.3 | 972.6 | 0.19 | 0.850(0.660-1.090) | 723.4 | 732.2 | 0.56 | 0.920(0.710-1.210) | 599.6 | 616 |
| rs26618 | Codominant | T/T | 238(0.547) | 122(0.439) | 79(0.462) |  | 1 | 948.3 | 971.2 |  | Ref | 723.5 | 736.7 |  | Ref | 600.7 | 621.3 |
|  |  | C/T | 165(0.379) | 136(0.489) | 76(0.444) | 0.003 | 1.610(1.180-2.210) |  |  | 0.084 | 1.390(0.960-2.010) |  |  | 0.469 | 0.870(0.580-1.300) |  |  |
|  |  | C/C | 32(0.074) | 20(0.072) | 16(0.094) | 0.517 | 1.230(0.670-2.240) |  |  | 0.216 | 1.510(0.780-2.890) |  |  | 0.562 | 1.260(0.610-2.590) |  |  |
|  | Dominant | T/T | 238(0.547) | 122(0.439) | 79(0.462) |  | 1 | 947.1 | 965.4 |  | Ref | 721.6 | 730.4 |  | Ref | 599.7 | 616.1 |
|  |  | C/T-C/C | 197(0.453) | 156(0.561) | 92(0.538) | 0.005 | 1.550(1.140-2.100) |  |  | 0.059 | 1.410(0.990-2.010) |  |  | 0.68 | 0.920(0.630-1.350) |  |  |
|  | Recessive | T/T-C/T | 403(0.926) | 258(0.928) | 155(0.906) |  | 1 | 955.1 | 973.4 |  | Ref | 724.5 | 733.3 |  | Ref | 599.2 | 615.6 |
|  |  | C/C | 32(0.074) | 20(0.072) | 16(0.094) | 0.95 | 0.980(0.550-1.760) |  |  | 0.42 | 1.300(0.690-2.440) |  |  | 0.4 | 1.350(0.670-2.690) |  |  |
|  | Overdominant | T/T-C/C | 270(0.621) | 142(0.511) | 95(0.556) |  | 1 | 946.8 | 965 |  | Ref | 723 | 731.8 |  | Ref | 599.1 | 615.5 |
|  |  | C/T | 165(0.379) | 136(0.489) | 76(0.444) | 0.004 | 1.570(1.160-2.130) |  |  | 0.14 | 1.310(0.910-1.870) |  |  | 0.38 | 0.840(0.570-1.240) |  |  |
|  | Log-additive | --- | --- | --- | --- | 0.026 | 1.320(1.030-1.670) | 950.2 | 968.4 | 0.069 | 1.290(0.980-1.690) | 721.8 | 730.7 | 0.97 | 1.010(0.740-1.370) | 599.9 | 616.3 |
| rs26653 | Codominant | C/C | 127(0.292) | 69(0.248) | 42(0.246) |  | 1 | 954 | 976.8 |  | Ref | 725.7 | 738.9 |  | Ref | 601.5 | 622 |
|  |  | G/C | 210(0.483) | 152(0.547) | 90(0.526) | 0.118 | 1.360(0.940-1.950) |  |  | 0.234 | 1.300(0.850-1.990) |  |  | 0.907 | 0.970(0.610-1.540) |  |  |
|  |  | G/G | 98(0.225) | 57(0.205) | 39(0.228) | 0.761 | 1.090(0.700-1.690) |  |  | 0.476 | 1.200(0.720-2.000) |  |  | 0.682 | 1.130(0.640-1.980) |  |  |
|  | Dominant | C/C | 127(0.292) | 69(0.248) | 42(0.246) |  | 1 | 953.3 | 971.5 |  | Ref | 723.8 | 732.6 |  | Ref | 599.9 | 616.3 |
|  |  | G/C-G/G | 308(0.708) | 209(0.752) | 129(0.754) | 0.17 | 1.270(0.900-1.790) |  |  | 0.25 | 1.270(0.840-1.900) |  |  | 0.97 | 1.010(0.650-1.570) |  |  |
|  | Recessive | C/C-G/C | 337(0.775) | 221(0.795) | 132(0.772) |  | 1 | 954.7 | 973 |  | Ref | 725.1 | 733.9 |  | Ref | 599.5 | 615.9 |
|  |  | G/G | 98(0.225) | 57(0.205) | 39(0.228) | 0.53 | 0.890(0.610-1.290) |  |  | 0.94 | 1.020(0.670-1.550) |  |  | 0.54 | 1.160(0.730-1.840) |  |  |
|  | Overdominant | C/C-G/G | 225(0.517) | 126(0.453) | 81(0.474) |  | 1 | 952.1 | 970.4 |  | Ref | 724.2 | 733 |  | Ref | 599.7 | 616.1 |
|  |  | G/C | 210(0.483) | 152(0.547) | 90(0.526) | 0.084 | 1.310(0.960-1.770) |  |  | 0.33 | 1.190(0.840-1.700) |  |  | 0.64 | 0.910(0.620-1.340) |  |  |
|  | Log-additive | --- | --- | --- | --- | 0.62 | 1.060(0.850-1.310) | 954.9 | 973.1 | 0.44 | 1.100(0.860-1.420) | 724.5 | 733.4 | 0.69 | 1.060(0.800-1.400) | 599.7 | 616.2 |
| rs3734016 | Codominant | C/C | 306(0.703) | 214(0.770) | 133(0.778) |  | 1 | 953.3 | 976.1 |  | Ref | 722.7 | 735.9 |  | Ref | 601.7 | 622.2 |
|  |  | C/T | 119(0.274) | 58(0.209) | 33(0.193) | 0.048 | 0.700(0.490-1.000) |  |  | 0.042 | 0.640(0.410-0.990) |  |  | 0.718 | 0.930(0.570-1.510) |  |  |
|  |  | T/T | 10(0.023) | 6(0.022) | 5(0.029) | 0.77 | 0.900(0.320-2.520) |  |  | 0.801 | 1.150(0.390-3.430) |  |  | 0.633 | 1.240(0.370-4.190) |  |  |
|  | Dominant | C/C | 306(0.703) | 214(0.770) | 133(0.778) |  | 1 | 951.5 | 969.8 |  | Ref | 721.6 | 730.5 |  | Ref | 599.9 | 616.3 |
|  |  | C/T-T/T | 129(0.297) | 64(0.230) | 38(0.222) | 0.057 | 0.720(0.500-1.010) |  |  | 0.062 | 0.680(0.450-1.030) |  |  | 0.86 | 0.960(0.610-1.520) |  |  |
|  | Recessive | C/C-C/T | 425(0.977) | 272(0.978) | 166(0.971) |  | 1 | 955.1 | 973.4 |  | Ref | 724.9 | 733.8 |  | Ref | 599.8 | 616.2 |
|  |  | T/T | 10(0.023) | 6(0.022) | 5(0.029) | 0.97 | 0.980(0.350-2.740) |  |  | 0.66 | 1.280(0.430-3.800) |  |  | 0.72 | 1.250(0.370-4.230) |  |  |
|  | Overdominant | C/C-T/T | 316(0.726) | 220(0.791) | 138(0.807) |  | 1 | 951.3 | 969.6 |  | Ref | 720.7 | 729.6 |  | Ref | 599.8 | 616.2 |
|  |  | C/T | 119(0.274) | 58(0.209) | 33(0.193) | 0.052 | 0.700(0.490-1.010) |  |  | 0.036 | 0.640(0.410-0.980) |  |  | 0.75 | 0.920(0.570-1.500) |  |  |
|  | Log-additive | --- | --- | --- | --- | 0.089 | 0.770(0.560-1.040) | 952.2 | 970.5 | 0.13 | 0.760(0.530-1.090) | 722.9 | 731.7 | 0.97 | 0.990(0.670-1.470) | 599.9 | 616.3 |
| rs2549782 | Codominant | T/T | 147(0.338) | 75(0.270) | 31(0.181) |  | 1 | 953.2 | 976 |  | Ref | 709.1 | 722.3 |  | Ref | 593.3 | 613.8 |
|  |  | G/T | 213(0.490) | 152(0.547) | 93(0.544) | 0.058 | 1.420(1.000-2.010) |  |  | 0.002 | 2.070(1.310-3.270) |  |  | 0.117 | 1.510(0.920-2.490) |  |  |
|  |  | G/G | 75(0.172) | 51(0.184) | 47(0.275) | 0.212 | 1.320(0.830-2.070) |  |  | 0.000042 | 2.970(1.750-5.060) |  |  | 0.006 | 2.370(1.320-4.240) |  |  |
|  | Dominant | T/T | 147(0.338) | 75(0.270) | 31(0.181) |  | 1 | 951.3 | 969.6 |  | Ref | 709.7 | 718.5 |  | Ref | 594.6 | 611 |
|  |  | G/T-G/G | 288(0.662) | 203(0.730) | 140(0.819) | 0.05 | 1.390(1.000-1.940) |  |  | 0.0001 | 2.310(1.490-3.570) |  |  | 0.022 | 1.730(1.070-2.780) |  |  |
|  | Recessive | T/T-G/T | 360(0.828) | 227(0.817) | 124(0.725) |  | 1 | 955.1 | 973.3 |  | Ref | 717.5 | 726.3 |  | Ref | 594 | 610.4 |
|  |  | G/G | 75(0.172) | 51(0.184) | 47(0.275) | 0.79 | 1.060(0.710-1.570) |  |  | 0.0056 | 1.820(1.200-2.760) |  |  | 0.015 | 1.770(1.120-2.800) |  |  |
|  | Overdominant | T/T-G/G | 222(0.510) | 126(0.453) | 78(0.456) |  | 1 | 952.6 | 970.8 |  | Ref | 723.7 | 732.5 |  | Ref | 599.9 | 616.3 |
|  |  | G/T | 213(0.490) | 152(0.547) | 93(0.544) | 0.11 | 1.280(0.950-1.740) |  |  | 0.23 | 1.240(0.870-1.770) |  |  | 0.93 | 0.980(0.670-1.450) |  |  |
|  | Log-additive | --- | --- | --- | --- | 0.14 | 1.180(0.950-1.470) | 953 | 971.3 | <0.0001 | 1.720(1.320-2.230) | 708.1 | 716.9 | 0.0033 | 1.540(1.150-2.060) | 591.3 | 607.7 |
| rs2548538 | Codominant | A/A | 142(0.326) | 87(0.313) | 33(0.193) |  | 1 | 956.9 | 979.7 |  | Ref | 711.5 | 724.7 |  | Ref | 589.2 | 609.8 |
|  |  | A/T | 218(0.501) | 139(0.500) | 89(0.520) | 0.819 | 1.050(0.740-1.470) |  |  | 0.014 | 1.760(1.120-2.760) |  |  | 0.032 | 1.810(1.110-2.950) |  |  |
|  |  | T/T | 75(0.172) | 52(0.187) | 49(0.286) | 0.584 | 1.120(0.720-1.760) |  |  | 0.00008 | 2.810(1.670-4.740) |  |  | 0.001 | 2.740(1.550-4.830) |  |  |
|  | Dominant | A/A | 142(0.326) | 87(0.313) | 33(0.193) |  | 1 | 955 | 973.3 |  | Ref | 713.9 | 722.7 |  | Ref | 590.1 | 606.5 |
|  |  | A/T-T/T | 293(0.674) | 191(0.687) | 138(0.807) | 0.7 | 1.070(0.770-1.470) |  |  | 0.0008 | 2.030(1.320-3.110) |  |  | 0.0033 | 2.060(1.290-3.270) |  |  |
|  | Recessive | A/A-A/T | 360(0.828) | 226(0.813) | 122(0.713) |  | 1 | 954.9 | 973.2 |  | Ref | 715.8 | 724.6 |  | Ref | 593.1 | 609.5 |
|  |  | T/T | 75(0.172) | 52(0.187) | 49(0.286) | 0.66 | 1.090(0.740-1.620) |  |  | 0.0022 | 1.930(1.270-2.920) |  |  | 0.0091 | 1.830(1.160-2.890) |  |  |
|  | Overdominant | A/A-T/T | 217(0.499) | 139(0.500) | 82(0.480) |  | 1 | 955.1 | 973.4 |  | Ref | 725 | 733.8 |  | Ref | 599.6 | 616 |
|  |  | A/T | 218(0.501) | 139(0.500) | 89(0.520) | 0.99 | 1.000(0.740-1.360) |  |  | 0.67 | 1.080(0.760-1.540) |  |  | 0.59 | 1.110(0.750-1.630) |  |  |
|  | Log-additive | --- | --- | --- | --- | 0.62 | 1.060(0.850-1.320) | 954.9 | 973.1 | 0.0001 | 1.670(1.290-2.170) | 709.6 | 718.4 | 0.0004 | 1.650(1.240-2.190) | 587.4 | 603.9 |
| rs2248374 | Codominant | G/G | 149(0.342) | 88(0.316) | 37(0.216) |  | 1 | 956.6 | 979.5 |  | Ref | 714.7 | 727.9 |  | Ref | 592.6 | 613.2 |
|  |  | G/A | 209(0.480) | 139(0.500) | 87(0.509) | 0.493 | 1.130(0.800-1.590) |  |  | 0.02 | 1.680(1.080-2.600) |  |  | 0.095 | 1.550(0.960-2.490) |  |  |
|  |  | A/A | 77(0.177) | 51(0.184) | 47(0.275) | 0.611 | 1.100(0.710-1.720) |  |  | 0.000474 | 2.460(1.470-4.100) |  |  | 0.005 | 2.360(1.350-4.130) |  |  |
|  | Dominant | G/G | 149(0.342) | 88(0.316) | 37(0.216) |  | 1 | 954.6 | 972.9 |  | Ref | 715.6 | 724.4 |  | Ref | 593.5 | 609.9 |
|  |  | G/A-A/A | 286(0.658) | 190(0.683) | 134(0.784) | 0.48 | 1.120(0.810-1.550) |  |  | 0.002 | 1.890(1.250-2.860) |  |  | 0.012 | 1.760(1.130-2.760) |  |  |
|  | Recessive | G/G-G/A | 358(0.823) | 227(0.817) | 124(0.725) |  | 1 | 955.1 | 973.4 |  | Ref | 718.2 | 727 |  | Ref | 594 | 610.4 |
|  |  | A/A | 77(0.177) | 51(0.184) | 47(0.275) | 0.9 | 1.030(0.690-1.520) |  |  | 0.0085 | 1.760(1.160-2.670) |  |  | 0.015 | 1.770(1.120-2.800) |  |  |
|  | Overdominant | G/G-A/A | 226(0.520) | 139(0.500) | 84(0.491) |  | 1 | 954.8 | 973.1 |  | Ref | 724.7 | 733.6 |  | Ref | 599.8 | 616.3 |
|  |  | G/A | 209(0.480) | 139(0.500) | 87(0.509) | 0.57 | 1.090(0.810-1.480) |  |  | 0.53 | 1.120(0.790-1.600) |  |  | 0.83 | 1.040(0.710-1.530) |  |  |
|  | Log-additive | --- | --- | --- | --- | 0.58 | 1.060(0.860-1.320) | 954.8 | 973.1 | 0.0005 | 1.570(1.220-2.020) | 712.8 | 721.6 | 0.0024 | 1.540(1.160-2.030) | 590.6 | 607.1 |
| rs2287988 | Codominant | A/A | 150(0.345) | 83(0.299) | 36(0.211) |  | 1 | 955.5 | 978.4 |  | Ref | 712.2 | 725.4 |  | Ref | 593.5 | 614 |
|  |  | G/A | 215(0.494) | 146(0.525) | 89(0.520) | 0.239 | 1.230(0.870-1.730) |  |  | 0.014 | 1.720(1.110-2.680) |  |  | 0.156 | 1.450(0.900-2.330) |  |  |
|  |  | G/G | 70(0.161) | 49(0.176) | 46(0.269) | 0.309 | 1.240(0.780-1.950) |  |  | 0.000114 | 2.740(1.630-4.610) |  |  | 0.007 | 2.300(1.300-4.050) |  |  |
|  | Dominant | A/A | 150(0.345) | 83(0.299) | 36(0.211) |  | 1 | 953.5 | 971.8 |  | Ref | 714.2 | 723.1 |  | Ref | 595 | 611.4 |
|  |  | G/A-G/G | 285(0.655) | 195(0.701) | 135(0.790) | 0.21 | 1.230(0.890-1.710) |  |  | 0.001 | 1.970(1.300-3.000) |  |  | 0.026 | 1.660(1.050-2.600) |  |  |
|  | Recessive | A/A-G/A | 365(0.839) | 229(0.824) | 125(0.731) |  | 1 | 955 | 973.2 |  | Ref | 716.3 | 725.1 |  | Ref | 593.9 | 610.3 |
|  |  | G/G | 70(0.161) | 49(0.176) | 46(0.269) | 0.68 | 1.090(0.730-1.630) |  |  | 0.003 | 1.920(1.260-2.930) |  |  | 0.014 | 1.790(1.130-2.840) |  |  |
|  | Overdominant | A/A-G/G | 220(0.506) | 132(0.475) | 82(0.480) |  | 1 | 954.4 | 972.6 |  | Ref | 724.8 | 733.6 |  | Ref | 599.9 | 616.3 |
|  |  | G/A | 215(0.494) | 146(0.525) | 89(0.520) | 0.38 | 1.140(0.840-1.550) |  |  | 0.56 | 1.110(0.780-1.580) |  |  | 0.94 | 0.980(0.670-1.450) |  |  |
|  | Log-additive | --- | --- | --- | --- | 0.28 | 1.130(0.910-1.410) | 953.9 | 972.2 | 0.0001 | 1.660(1.280-2.150) | 710.2 | 719 | 0.0039 | 1.510(1.140-2.010) | 591.6 | 608 |
| rs1056893 | Codominant | T/T | 161(0.370) | 88(0.316) | 39(0.228) |  | 1 | 954.7 | 977.5 |  | Ref | 711.4 | 724.7 |  | Ref | 594.7 | 615.3 |
|  |  | C/T | 205(0.471) | 138(0.496) | 86(0.503) | 0.226 | 1.250(0.890-1.750) |  |  | 0.012 | 1.730(1.130-2.660) |  |  | 0.149 | 1.450(0.910-2.320) |  |  |
|  |  | C/C | 69(0.159) | 52(0.187) | 46(0.269) | 0.155 | 1.370(0.870-2.140) |  |  | 0.000079 | 2.750(1.650-4.590) |  |  | 0.013 | 2.120(1.220-3.680) |  |  |
|  | Dominant | T/T | 161(0.370) | 88(0.316) | 39(0.228) |  | 1 | 952.9 | 971.1 |  | Ref | 713.5 | 722.3 |  | Ref | 595 | 611.5 |
|  |  | C/T-C/C | 274(0.630) | 190(0.683) | 132(0.772) | 0.13 | 1.280(0.930-1.760) |  |  | 0.0006 | 1.990(1.320-2.990) |  |  | 0.028 | 1.630(1.050-2.540) |  |  |
|  | Recessive | T/T-C/T | 366(0.841) | 226(0.813) | 125(0.731) |  | 1 | 954.3 | 972.6 |  | Ref | 715.9 | 724.7 |  | Ref | 595.2 | 611.6 |
|  |  | C/C | 69(0.159) | 52(0.187) | 46(0.269) | 0.37 | 1.200(0.810-1.790) |  |  | 0.0024 | 1.950(1.280-2.990) |  |  | 0.03 | 1.660(1.050-2.630) |  |  |
|  | Overdominant | T/T-C/C | 230(0.529) | 140(0.504) | 85(0.497) |  | 1 | 954.5 | 972.8 |  | Ref | 724.6 | 733.5 |  | Ref | 599.9 | 616.3 |
|  |  | C/T | 205(0.471) | 138(0.496) | 86(0.503) | 0.45 | 1.120(0.830-1.520) |  |  | 0.48 | 1.140(0.800-1.620) |  |  | 0.86 | 1.030(0.700-1.520) |  |  |
|  | Log-additive | --- | --- | --- | --- | 0.13 | 1.180(0.950-1.470) | 952.8 | 971.1 | 0.0001 | 1.660(1.290-2.140) | 709.5 | 718.3 | 0.0075 | 1.450(1.100-1.920) | 592.7 | 609.2 |

Note: The statistical significant threshold was set at P<0.0045 after Bonferroni correction

*The P-value, OR and 95%CIs of pairs comparison between ITTB and control were calculated on the basis of the logistic regression model adjusted by age

**The P-value, OR and 95%CIs of pairs comparison between ITTB and RTB were calculated on the basis of the logistic regression model adjusted by gender
